# Development of a free radical scavenging bacterial consortium to mitigate oxidative stress in cnidarians

**DOI:** 10.1111/1751-7915.13877

**Published:** 2021-07-14

**Authors:** Ashley M. Dungan, Dieter Bulach, Heyu Lin, Madeleine J. H. van Oppen, Linda L. Blackall

**Affiliations:** ^1^ School of Biosciences The University of Melbourne Melbourne Vic. Australia; ^2^ Melbourne Bioinformatics The University of Melbourne Melbourne Vic. Australia; ^3^ School of Earth Sciences The University of Melbourne Melbourne Vic. Australia; ^4^ Australian Institute of Marine Science Townsville Qld Australia

## Abstract

Corals are colonized by symbiotic microorganisms that profoundly influence the animal’s health. One noted symbiont is a single‐celled alga (in the dinoflagellate family *Symbiodiniaceae*), which provides the coral with most of its fixed carbon. Thermal stress increases the production of reactive oxygen species (ROS) by *Symbiodiniaceae* during photosynthesis. ROS can both damage the algal symbiont’s photosynthetic machinery and inhibit its repair, causing a positive feedback loop for the toxic accumulation of ROS. If not scavenged by the antioxidant network, excess ROS may trigger a signaling cascade ending with the coral host and algal symbiont disassociating in a process known as bleaching. We use *Exaiptasia diaphana* as a model for corals and constructed a consortium comprised of *E. diaphana*–associated bacteria capable of neutralizing ROS. We identified six strains with high free radical scavenging (FRS) ability belonging to the families *Alteromonadaceae*, *Rhodobacteraceae*, *Flavobacteriaceae* and *Micrococcaceae*. In parallel, we established a consortium of low FRS isolates consisting of genetically related strains. Bacterial whole genome sequences were used to identify key pathways that are known to influence ROS.

## Graphical abstract

The field of coral microbiome engineering is in its infancy and is currently limited by a lack of definitive information about the functional roles of cnidarian microbiome members. Outlined in this manuscript is the start of a complex process to identify, evaluate, and select durable and useful candidate consortium members that may buffer the coral host against climate warming. We identified six diverse bacterial strains with high free radical scavenging (FRS) ability and six conspecific/congeneric low FRS strains with a view to including them in an inoculum to mitigate the effects of thermal stress in cnidarians.

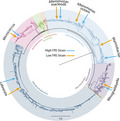

## Introduction

Coral reefs are among the most biologically and economically valuable ecosystems on Earth (Cesar *et al*., 2003; Alder *et al*., 2006; Fisher *et al*., 2015). While they cover less than 0.1% of the ocean floor (Spalding and Grenfell, 1997), coral reefs support economic activities relating to fisheries, tourism, pharmaceuticals and coastal development with a global value of $8.9 trillion “international $” per year (de Groot *et al*., 2012). Corals and other reef organisms have been dying, largely due to anthropogenic influences such as climate change (Hughes *et al*., 2017; Stuart‐Smith *et al*., 2018), which has led to an increased frequency, intensity and duration of summer heat waves that cause coral bleaching (Hughes *et al*., 2018; Hoegh‐Guldberg *et al*., 2019).

The coral holobiont, which is the sum of the coral animal and its associated microbiota, including algae, fungi, protozoans, bacteria, archaea and viruses (Rohwer *et al*., 2002), is an ecosystem engineer. By secreting a calcium carbonate skeleton, scleractinian corals form the literal foundation of the coral reef ecosystem. The success of corals to survive and build up reefs over thousands of years (Devlin‐Durante *et al*., 2016) is tightly linked to their obligate yet fragile symbiosis with endosymbiotic dinoflagellates of the family *Symbiodiniaceae* (Glynn, 1996).

Intracellular *Symbiodiniaceae* translocate photosynthetically fixed carbon to the coral host (Muscatine and Porter, 1977; Tremblay *et al*., 2014) in exchange for inorganic nutrients and location in a high light environment with protection from herbivory (Venn *et al*., 2008; Yellowlees *et al*., 2008). During periods of thermal stress, the relationship between the coral host and their *Symbiodiniaceae* can break down, resulting in a separation of the partners and significantly, a fixed carbon shortage for the host (Radecker *et al*., 2021). This phenomenon, ‘coral bleaching’, is devastating to the host and detrimental to the reef system. The ecosystem‐wide effects of bleaching on the coral include reduced skeletal growth and reproductive activity, a lowered capacity to shed sediments, and an inability to resist invasion of competing species and diseases. Severe and prolonged bleaching can impact coral health and ultimately cause colony death, sequentially observed as diminished reef growth, the transformation of reef‐building communities to alternate, non‐reef building community types, bioerosion and ultimately the disappearance of reef structures (Glynn, 1996).

There are several hypotheses detailing the mechanisms driving bleaching (Weis, 2008; Cunning and Baker, 2012; Wiedenmann *et al*., 2012; Wooldridge, 2013), with a common theme being the overproduction and toxic accumulation of reactive oxygen species (ROS). ROS production is a normal part of the physiology and functioning of organisms, including as a product of photosynthesis. The light triggered splitting of water molecules in the oxygen evolving complex (OEC), and subsequent transfer of electrons from photosystem II (PSII) to photosystem I (PSI) generates ROS in chloroplasts (Trubitsin *et al*., 2014). Additionally, photodamage, particularly to PSII, is a normal part of the photosynthetic light cycle, with mechanisms available to efficiently repair this damage (Aro *et al*., 1993). Heat affects the fluidity and integrity of the thylakoid membrane, disturbing PSI, PSII and the OEC, while simultaneously inducing the production of ROS (Farooq *et al*., 2016). Elevated intracellular ROS levels can both increase damage to (Mathur *et al*., 2014) and inhibit the repair of the D1 protein in the PSII apparatus (Warner *et al*., 1999; Nishiyama *et al*., 2006), and, in a positive feedback mechanism, excess ROS is generated. Once generated, ROS can trigger the oxidation of essential photosynthetic molecules, such as thylakoid membranes (Tchernov *et al*., 2004) and enzymes of the Calvin‐Benson cycle (Lesser and Farrell, 2004), thereby interfering with the supply of fixed carbon to the holobiont (Lesser, 2004, 2004).

It is hypothesized that ROS, specifically hydrogen peroxide (H_2_O_2_), can transfer from the algal symbiont to the surrounding host cell (Szabó *et al*., 2020). ROS can also be generated from actively growing bacteria (Zinser, 2018; Hansel and Diaz, 2021), these include bacterial coral symbionts (Zhang *et al*., 2016). Excess ROS may damage both the host and symbiont cellular machinery. Once damaged, *Symbiodiniaceae* are no longer able to maintain their role in the relationship with corals and separate from the host tissue via *in situ* degradation or exocytosis (Weis, 2008). Thus, any mechanism that might neutralize ROS in host or *Symbiodiniaceae* cells could reduce coral bleaching.

Microbiome engineering through the addition of a selection of beneficial bacteria has been proposed as a strategy to facilitate adaptation to changing environmental conditions by enhancing the coral holobiont with the metabolic capabilities of the introduced bacteria (van Oppen *et al*., 2015; Damjanovic *et al*., 2017; Peixoto *et al*., 2017; van Oppen *et al*., 2017; Damjanovic *et al*., 2019; Epstein *et al*., 2019, 2019,2019, 2019; van Oppen and Blackall, 2019; Peixoto *et al*., 2021). The differences in the bacterial community composition and stability of healthy and thermally stressed corals (Vega Thurber *et al*., 2009; Mouchka *et al*., 2010; Sunagawa *et al*., 2010; Littman *et al*., 2011; Epstein *et al*., 2019, 2019,2019, 2019; Pootakham *et al*., 2019) and the coral model *Exaiptasia diaphana* (Plovie, 2010; Ahmed *et al*., 2019; Hartman *et al*., 2020) demonstrate an adaptation of the host‐associated microbiome to changing external environments and support the potential utility of microbiome engineering in cnidarian health. In addition, a disruption to the bacterial community of *Pocillopora damicornis* with antibiotic treatment diminished the resilience of the holobiont during thermal stress, whereas intact microbial communities conferred resilience to thermal stress and increased the rate of holobiont recovery after bleaching events (Gilbert *et al*., 2012). The relative stability of coral‐associated bacterial communities has also been linked to coral heat tolerance; for instance, the bacterial community of heat‐sensitive *Acropora hyacinthus* corals shifted when transplanted to thermal stress conditions, whereas heat‐tolerant *A. hyacinthus* corals harbored a stable bacterial community (Ziegler *et al*., 2017).

In recent years, researchers have begun to explore microbiome engineering in corals and *E. diaphana*. To inhibit the progression of white pox disease, caused by pathogenic *Serratia marcescens*, an *Alphaproteobacteria* cocktail containing several *Marinobacter* spp. was applied to *E*. *diaphana* (Alagely *et al*., 2011); these introduced strains were able to inhibit both biofilm formation and swarming of *S. marcescens,* which halted disease progression. The *Marinobacter*‐based inoculum was deemed effective as anemones exposed to both the cocktail and pathogen survived after seven days, while anemones in the *S*. *marcescens* control treatment died. A bacterial consortium native to the coral *Mussismilia harttii* was selected to degrade water‐soluble oil fractions (dos Santos *et al*., 2015). This bioremediation strategy reduced the negative impacts of oil on *M. harttii* health and accelerated the degradation of petroleum hydrocarbons (dos Santos *et al*., 2015). Coral microbiomes have also been manipulated through addition of a consortium of native or seawater‐derived bacteria to the surface of *P. damicornis* to mitigate the effects of thermal stress (Rosado *et al*., 2018). The results from this study suggest the consortium was able to partially mitigate coral bleaching.

Our goal was to identify bacterial strains suitable for use in a microbiome engineering approach to mitigate the effects of thermal stress in *E. diaphana*. Given the potential role of ROS in the bleaching process and the prevalence of bacteria in and on hosts (Lesser *et al*., 2004; Work and Aeby, 2014) and intracellular *Symbiodiniaceae* (Ainsworth *et al*., 2015; Maire *et al*., 2021), our focus was to select diverse *E*. *diaphana*–sourced bacterial isolates with an extracellular free radical scavenging (FRS) phenotype.

## Results

### Diversity of culturable bacteria associated with *E. diaphana*

**Fig. 1:**
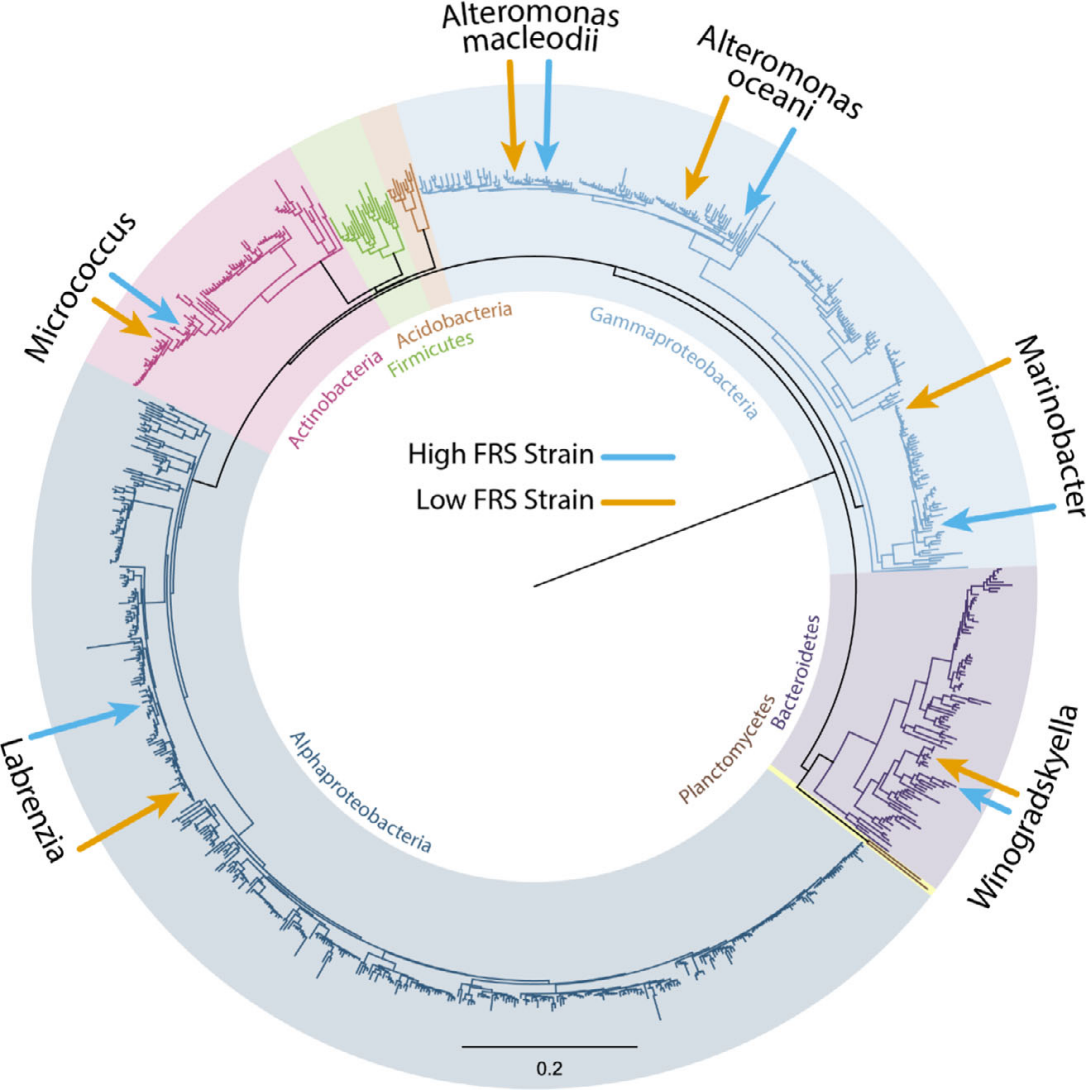
Neighbor‐Joining tree showing an overview of the phylogenetic relationship between the 842 *E. diaphana*–associated bacterial isolates inferred using partial 16S rRNA sequences. These isolates covered six phyla indicated by shading over the tree with *Proteobacteria* split into the classes *Gammaproteobacteria* and *Alphaproteobacteria*. The positions of selected strains are highlighted by arrows with blue arrows indicating the high FRS strains and orange arrows indicating low FRS strains.

A total of 842 isolates were obtained from four genotypes of Great Barrier Reef (GBR)–sourced *E*. *diaphana*. There were no significant differences in bacterial colony forming units (CFUs) between the four genotypes, regardless of growth medium, with 5.9–10.3 × 10^3^ CFUs per anemone on Reasoner's 2A agar (R2A) and 6.3–10.4 × 10^3^ CFUs per anemone on marine agar (MA) (*P* > 0.05). Partial 16S rRNA gene sequences (˜ 1000 bp) were used to identify the closest matches from GenBank using the Basic Local Alignment Search Tool (Blastn). In total there were 109 species in 64 genera, 27 families and six phyla (Fig. 1). The most abundant genera (Table 1) were *Alteromonas, Labrenzia* and *Ruegeria*. Gram‐positive bacteria comprised 23 species, including *Microbacterium* (31 isolates) and *Micrococcus* (28 isolates). Eight genera were found to be associated with all four genotypes (Table 1); these eight genera made up 59.4% of all *E*. *diaphana*–associated bacterial isolates.

**Table 1:** Bacterial genera associated with all four genotypes of GBR‐sourced *E. diaphana* (AIMS1‐4).

Genus	Class	No. of species	AIMS1	AIMS2	AIMS3	AIMS4	Total isolates^a^
*Alteromonas*	*Gamma‐proteobacteria*	6	10	52	24	30	116
*Labrenzia*	*Alpha‐proteobacteria*	4	11	10	26	38	85
*Marinobacter*	*Gamma‐proteobacteria*	4	7	25	10	13	55
*Muricauda*	*Flavobacteriia*	1	16	11	10	5	42
*Roseovarius*	*Alpha‐proteobacteria*	3	9	12	5	6	32
*Ruegeria*	*Alpha‐proteobacteria*	3	29	8	5	39	81
*Shimia*	*Alpha‐proteobacteria*	2	2	3	9	40	54
*Vibrio*	*Gamma‐proteobacteria*	3	3	7	31	15	56

^a^ 521 out of the 842 isolates obtained from the four *E. diaphana* genotypes.

### Bacterial consortium selection

**Fig. 2:**
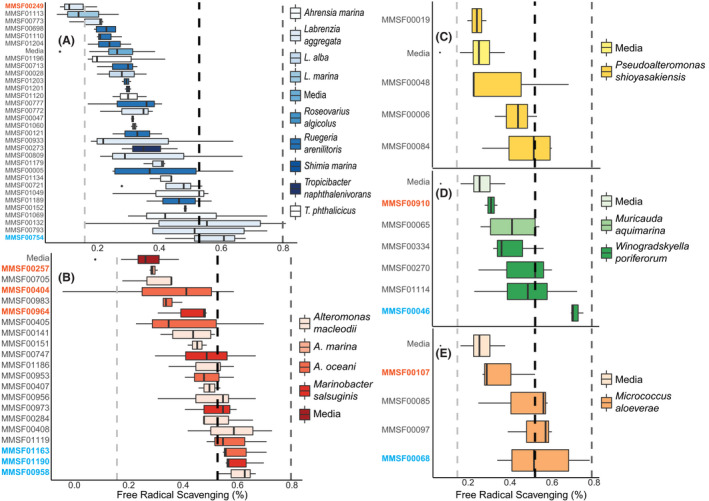
Quantitative FRS ability of *E. diaphana*–associated bacterial isolates, separated by Family. Families with high relative abundance among all cultured bacteria (*Rhodobacteraceae* – A, *Alteromonadaceae* – B, *Pseudoalteromonadaceae* – C, *Flavobacteraceae* – D, and *Micrococcaceae* – E) were separately analyzed to identify strains with a high FRS ability (blue font) and a corresponding conspecific or congeneric strain with a low FRS ability (orange font). In each panel, the light dashed vertical line on the left represents the mean FRS of a 0.025% (w/v) ascorbic acid standard, the middle dark dashed vertical line is the mean FRS for 0.05% (w/v) ascorbic acid standard, and the far‐right dashed line is the mean FRS of the 0.075% (w/v) ascorbic acid standard.

A high extracellular FRS phenotype was the primary selection criteria in selecting *E. diaphana*–sourced bacterial isolates for inclusion in the consortium. The FRS phenotype was measured using the stable free radical 2,2‐diphenyl‐1‐picrylhydrazyl (DPPH), which is reduced in the presence of an antioxidant molecule, undergoing a color change from a violet to a colorless solution. Of the original 842 isolates, 709 were qualitatively screened for their ability to scavenge exogenous free radicals, divided into positive (144), weakly positive (121) and negative (444). Ninety‐eight strains representing eight families and 18 genera were then quantitatively assessed for FRS. There was no clear pattern of FRS capacity at the family level (Fig. 2) with strain‐specific responses evident. Consortium members were selected by choosing *E. diaphana*–associated bacterial isolates, where conspecific or congeneric pairs of strains displayed a high and low FRS ability (Fig. 2; Table 2). Of the 12 selected bacterial isolates, seven were catalase positive and five were catalase negative (Table 2). In each consortium set (i.e., high or low FRS strains), none of the selected isolates showed antagonistic activity against one other as evidenced by the absence of any zone of inhibition and growth from each combination of isolates on a plate. Growth curves show that after 48 h at 37°C, each selected isolate was in the stationary phase of growth (Figs S1–S12).

**Table 2:** Overview of high S bacterial strains and conspecific/congeneric low FRS strains. All sequence data can be found under BioProject^a^ PRJNA574193. References to each selected strain at the Genus level are identified in the last three columns.

Strain	Bacteria species (Phylum or Class)	FRS^a^ (% ± SE)	High versus low FRS^c^	Strain versus growth medium^c^	Catalase	*E. diaphana* literature	Coral literature	Probiotic literature
MMSF01163 (*n* = 3)	*Alteromonas oceani* (*Gammaproteobacteria*)	61.7 ± 5.2	0.065	**0.004**	Negative	Binsarhan (2016), Röthig *et al*. (2016), Herrera *et al*. (2017)	Chiu *et al*. (2012), Ceh *et al*. (2013), Röthig *et al*. ( 2017), Damjanovic *et al*. (2019)	Riquelme *et al*. (1997), Kesarcodi‐Watson *et al*. (2010), Hai (2015), Haryanti *et al*. (2017)
MMSF00404 (*n* = 4)		35.5 ± 13.7		0.571	Negative			
MMSF00958 (*n* = 3)	*Alteromonas macleodii* (*Gammaproteobacteria*)	62.0 ± 4.2	0.175	**0.017**	Negative			
MMSF00257 (*n* = 3)		30.3 ± 0.9		0.431	Positive			
MMSF00132 (*n* = 8)	*Labrenzia aggregata* (Biebl *et al*., 2007) (*Alphaproteobacteria*)	53.6 ± 8.7	**0.016**	0.101	Negative	Binsarhan (2016)	Littman *et al*. (2011), Chiu *et al*. (2012)	Mancuso *et al*. (2015)
MMSF00249 (*n* = 3)		14.0 ± 3.6		0.103	Positive			
MMSF01190 (*n* = 3)	*Marinobacter salsuginis* (*Gammaproteobacteria*)	62.0 ± 4.5	**0.041**	**< 0.0001**	Negative	Binsarhan (2016), Brown *et al*. (2017), Herrera *et al*. (2017)	Littman *et al*. (2009), Sharp *et al*. (2012), Röthig *et al*. (2017)	Alagely *et al*. (2011)
MMSF00964 (*n* = 3)		43.7 ± 5.8		**0.020**	Positive			
MMSF00068 (*n* = 6)	*Micrococcus luteus* (*Actinobacteria*)	56.3 ± 7.3	0.210	**0.002**	Positive	Binsarhan (2016)	Kellogg *et al*. (2013)	El‐Rhman *et al*. (2009), Osman *et al*. (2010), Hai (2015)
MMSF00107 (*n* = 3)	*Micrococcus yunnanensis* (*Actinobacteria*)	38.0 ± 8.0		0.306	Positive			
MMSF00046 (*n* = 3)	*Winogradskyella poriferorum* (Lau *et al*., 2005) (*Bacteroidetes*)	73.3 ± 1.9	0.284	**0.010**	Negative	^b^	dos Santos *et al*. (2015), Franco *et al*. (2018)	NA
MMSF00910 (*n* = 3)		36.0 ± 3.5		0.174	Negative			

^a^ Mean R2A FRS (% ± SE) was 27.2 ± 2.3% (*n* = 12), and it was catalase negative.

^b^ While there are no instances of *Winogradskyella* specifically identified in current *E. diaphana* literature, there are several *Flavobacteriaceae* that are not resolved to the genus level from metabarcoding data (Plovie, 2010; Röthig *et al*., 2016; Herrera *et al*., 2017; Ahmed *et al*., 2019).

^c^ Indicates *P* values for pairwise comparisons from respective one‐way analysis of variance (Tukey HSD) or Kruskal‐Wallis rank sum test (Dunn test) with bold values representing significant differences.

### Comparative genomics

As part of the characterization of the 12 isolates (six high FRS and six conspecific or congeneric low FRS isolates), draft genome sequences were assembled and analyzed. A summary of the data and metrics for the draft genome sequences is presented in Table S1. The diversity of the six pairs of isolates is indicated by the %G + C range (35% to 72%) and genome size (2.4 Mb to 6.8 Mb). Each isolate pair was classified as the same species, according to 16S rRNA gene sequence identity, except the *Micrococcus* stains. Isolates MMSF00068 (high FRS strain) and MMSF00107 (low FRS strain) are classified as *Micrococcus luteus* and *M*. *yunnanensis*, respectively.

Core genome comparison provides an overview of the genetic relationship between the conspecific or congeneric isolate pairs. A wide range of genome variation between strain pairs was observed, which ranged from ˜ 190 000 single nucleotide polymorphism (SNP) differences between the *Alteromonas oceani* strains to fewer than five core genome SNPs between the *Labrenzia* *aggregata* isolates (Table S2). It should be noted that core genome comparison cannot be used to establish isolate identity with differences in the accessory genome content necessarily not included. In our study, this is best illustrated with the *Winogradskyella* *poriferorum* isolates MMSF00046 and MMSF00910; the core genomes differ by fewer than ten pairwise SNPs, but there are accessory genome differences.

### Genes of interest

The annotated genome sequences of each selected candidate consortium member were searched for key genes relevant to extracellular ROS scavenging capabilities (Tables S3 and S4). Dimethylsulfoniopropionate (DMSP) cleavage to dimethylsulfide (DMS) was identified by presence of one or more of the DMSP lyase genes; *dddP*, *dddD*, *dddL*, *dddW* and *dddQ*. Only *L*. *aggregata* strains contained DMSP lyase genes (*dddP* and *dddL*) in their whole genome sequences. DMSP biosynthesis was identified by the presence of *dsyB*, which is the only described gene for an enzyme in the DMSP biosynthesis pathway. The *cobP* gene was used as an indicator for the presence of the dynamic vitamin B_12_ pathway, which contains 27 genes (Table S4). Again, only the *L. aggregata* isolates contained *cobP*. Catalase positive strains were identified by the presence of *katG*; all high and low FRS strains contained *katG* except the *Micrococcus* spp. strains, in which *katA* and *katE* were detected.

### 16S rRNA gene copy number

The 16S rRNA gene copy numbers of the 12 draft genomes were estimated using a read depth approach (Table S1). The copy numbers were similar within pairs of isolates, in which the pair of *A*. *macleodii* isolates (MMSF00257 and MMSF00958) contained the most copies (5.15 and 4.79, respectively), corresponding with copy numbers in a published closed genome of *A*. *macleodii* (Gonzaga *et al*., 2012). *W*. *poriferorum* isolates (MMSF00046 and MMSF00910) contained the fewest copies (1.03 and 0.77, respectively).

## Discussion

The 842 *E*. *diaphana* bacterial isolates reported here comprise 109 species from 64 genera and six phyla. Using metabarcoding, studies of microbiomes associated with *E*. *diaphana* have revealed a similar diversity at the phylum level for *E*. *diaphana* sourced from the GBR (Hartman *et al*., 2020; Dungan *et al*., 2021), Hawaii (strain H2; Herrera *et al*., 2017), Pacific and Caribbean (Brown *et al*., 2017), Atlantic (strain CC7; Röthig *et al*., 2016) and Red Sea (Ahmed *et al*., 2019), as well as stony corals (Blackall *et al*., 2015). Thus, our culture collection of *E. diaphana* bacterial isolates captures the diversity of the *E. diaphana*–associated microbiome. The broader culture collection contains bacteria with a wide range of FRS capacity, and our candidate consortia comprise greater bacterial diversity than others (Alagely *et al*., 2011; Rosado *et al*., 2018).

The consistent and frequent reporting of our selected bacterial genera in *E*. *diaphana* and coral studies (Table 2) suggests these bacteria likely have key functions in cnidarian holobionts. Among these potential functions are the production and secretion of antioxidants and other reducing agents. Antioxidants of interest include DMSP and the breakdown of DMSP to other antioxidants (Sunda *et al*., 2002). *L*. *aggregata* have been reported to produce DMSP in the absence of any methylated sulfur compounds with *dsyB* identified as the first DMSP biosynthesis gene in any organism (Curson *et al*., 2017). *dsyB* was found in the genome sequences of both high and low FRS *L. aggregata* strains (Table S3). Many of the *E. diaphana*–sourced bacterial species, in particular, bacteria related to our selected isolates, have been implicated in the degradation of DMSP to DMS (*Alteromonas* spp., Raina *et al*., 2009; *Labrenzia* spp., Hatton *et al*., 2012). The *dddP* gene which encodes for the enzyme responsible for cleaving DMSP to DMS, was used as an indicator of a DMSP degradation genotype. Using the products identified in the Prokka annotation of each of the genomes, only the *L*. *aggregata* isolates contained genes responsible for DMSP degradation (Table S3).

Carotenoids are among the strongest antioxidants and are highly reactive against ROS and other free radicals (Fiedor *et al*., 2005; Asker *et al*., 2007; Shindo *et al*., 2007; Fiedor and Burda, 2014; Flórez *et al*., 2015). Carotenoids are lipid‐soluble pigments, and in bacteria they give an orange‐yellow hue to colonies. Two of the five selected genera produce orange/yellow colonies (*Winogradskyella*, *Micrococcus*), and there is evidence of carotenoid production by marine *Flavobacteriaceae* (Shindo *et al*., 2007; Gammone *et al*., 2015) and *Micrococcus* (Mohana *et al*., 2013). A marine *Flavobacteriaceae* (strain GF1) was found to produce the potent antioxidant carotenoid zeaxanthin that protected *Symbiodiniaceae* from thermal and light stress (Motone *et al*., 2020).

Vitamin B_12_ is a cofactor required for the biosynthesis of the amino acid methionine, a fundamental component of every protein, and in diverse metabolic pathways including generation of the antioxidants glutathione and DMSP (Croft *et al*., 2005). Vitamin B_12_ is synthesized by many heterotrophic bacteria (Raux *et al*., 2000). Genomic evidence suggests that *Symbiodiniaceae* have lost the capacity to synthesize vitamin B_12_ (Matthews *et al*., 2020), which is in agreement with another work showing that free‐living *Symbiodiniaceae* rely on bacterial symbionts for this important cofactor (Agostini *et al*., 2009). The genes involved in the biosynthesis of vitamin B_12_ have been found in coral‐associated bacteria (Robbins *et al*., 2019), specifically *L. aggregata* cultured from the Caribbean coral, *Orbicella faveolata* (Smith, 2018). Eighteen genes, including *cobP* (Raux *et al*., 2000), are present in each *L. aggregata* isolate (MMSF00132 and MMSF00249), suggesting both are capable of vitamin B_12_ biosynthesis. None of the other sequenced isolated had genes related to vitamin B_12_ biosynthesis, except in the *Marinobacter salsuginis* isolates, where *cobO* was detected, strongly indicating that none of the remaining ten isolates were capable of vitamin B_12_ biosynthesis (Table S4).

Bacteria have developed highly specific mechanisms to protect themselves against oxidative stress, in particular using enzymatic antioxidants such as catalase, peroxidase, and superoxide dismutase (Imlay, 2018). It has been suggested that increasing the *in hospite* concentration of catalase in the coral holobiont by the application of a bacterial consortium with catalase‐positive organisms could possibly minimize the impact of thermal stress by neutralizing H_2_O_2_ (Peixoto *et al*., 2017). Here we tested isolates for catalase production using a standard H_2_O_2_ assay (Taylor and Achanzar, 1972). Catalase participates in cellular antioxidant defense by enzymatically breaking down H_2_O_2_ to H_2_O and O_2_. The enzymes involved are hydroperoxidase I (HPI, encoded by *katG*), a bifunctional enzyme with both catalase and peroxidase activities expressed during aerobic growth, and hydroperoxidase II (HPII, *katE*), a monofunctional enzyme with catalase activity expressed during stationary phase (von Ossowski *et al*., 1991; Cabiscol *et al*., 2000). Each of the isolates had at least one *katG* or *katE* (Table S3). The lack of correlation between genotype and phenotype in relation to catalase activity may be associated with the culture conditions used, the concentration of H_2_O_2_ used in this assay [chosen to replicate previous studies (Rosado *et al*., 2018)], or undetected, as not all ROS scavenging activity can be observed by bubble formation.

The selected isolates were chosen from a highly diverse pool of *E. diaphana*–sourced isolates. While the selected isolates are phylogenetically diverse, other potentially promising beneficial bacteria in the culture collection were omitted based on our selection criteria. For example, *Ruegeria* spp. have been reported to breakdown DMSP and participate in denitrification (Smith, 2018). *Muricauda* isolates were found to have high FRS abilities, but were excluded from the consortium due to sporadic growth on the medium used in the study. *Muricauda* isolates have genes for denitrification (Smith, 2018), can breakdown DMSP (Hatton *et al*., 2012) and produce potent carotenoids (Prabhu *et al*., 2014) that can mitigate thermal and light stresses in *Symbiodiniaceae* cultures (Motone *et al*., 2020). Both *Ruegeria* and *Muricauda* should be included in future consortium evaluations.

Interactions among members of the microbiota associated with marine animals are undoubtedly complex. Results presented in this manuscript show that pure cultured bacteria from *E*. *diaphana* can scavenge free radicals in a strain‐specific fashion, reinforcing our approach of phenotypic selection. While there has been a surge of research and interest in microbial ROS production (Rose *et al*., 2008; Sutherland *et al*., 2019; Hansel and Diaz, 2021), there is a lack of data on ROS scavenging by specific microbial isolates. Therefore, the finding that *E. diaphana*–sourced bacterial isolates have a range of capacities to quench the stable N radical associated with DPPH improves our understanding of the overall extracellular FRS potential of these cultures. However, while ROS are all free radicals, there are many other types of free radicals whose activity our assay would have captured. Thus, a high FRS phenotype cannot perfectly translate to high ROS scavenging. Ultimately the role of these bacteria in coral/anemone physiology and health and the ROS dynamics within the cnidarian holobiont remains unknown.

The 12 candidate isolates making up the consortium were grown for 48 h while these cells were in stationary phase (Figs S1–S12). The life stage can play a critical role in FRS behavior with stationary phase *Roseobacter* isolates known to be more efficient in degrading superoxide (Hansel *et al*., 2019). We recognize that this may not be representative of the extracellular FRS for actively growing/respiring bacteria cells *in hospite*.

Inoculation with high FRS strains could be beneficial to the host under high oxidative stress conditions, such as those that potentially contribute to coral bleaching. While excessive ROS have been implicated in the onset of oxidative stress and subsequent bleaching of corals, this link has yet to be definitively shown and the role of ROS in coral health is nuanced and complex (Krueger *et al*., 2015; Nielsen *et al*., 2018) as ROS also provides beneficial benefits to the host (Hansel and Diaz, 2021). Future research should explore other beneficial bacterial functions such as alternative carbon fixation mechanisms, nitrogen fixation, and quorum quenching to disrupt cell‐to‐cell signalling of pathogens (Peixoto *et al*., 2021).

The field of coral microbiome engineering is in its infancy and is currently limited by a lack of definitive information about the functional roles of cnidarian microbiome members (van Oppen and Blackall, 2019; Blackall *et al*., 2020). Information that would aid development includes determination of bacterial phenotypes that are beneficial to the host, such as extracellular free radical scavenging. Outlined here is the start of a complex process to identify, evaluate and select durable and useful candidate consortium members that may buffer the coral host against climate warming. This consortium can be explored in the future to determine whether their addition provides a benefit to the host (i.e., are probiotics) and the underlying mechanism. Conspecific or congeneric pairs of bacteria provide an opportunity to determine the genetic basis for measured phenotypic differences between the pairs.

## Experimental procedures

### Isolation of bacterial isolates

*Exaiptasia diaphana* of GBR origin were maintained in the laboratory at 26°C (Dungan *et al*., 2020) and used to establish a bacterial isolate culture collection. Sixteen individuals from each of four *E. diaphana* genotypes (AIMS1‐4) were collected using sterile disposable pipets and gently transferred to filter‐sterilized (0.2 µm) reverse osmosis (RO) water reconstituted Red Sea Salt™ (Red Sea; RSS) at ˜ 34 parts per thousand (ppt) salinity (fRSS). After 30 min, each anemone was transferred to a sterile glass homogenizer with 1 ml of fRSS. Each homogenate was used to prepare serial dilutions from 10^−1^ to 10^−4^. From each dilution, 50 µl was spread plate–inoculated onto three replicate plates each of MA (Difco™ Marine Agar 2216, BD, Sparks, MD, USA) and R2A (R2A Agar CM0906, Oxoid, Oxoid Ltd., Basingstoke, Hampshire, England) supplemented with 40 g l^−1^ RSS and incubated at 26˚C. After one week, colony counts were completed. Individual colonies were sub‐cultured to purification from plates with < 100 colonies isolation medium. Single colonies (purified bacterial isolates) were resuspended individually in 40% glycerol, aliquoted into 1.2 ml cryotubes and stored at −80°C for long‐term preservation.

### Identification of *E. diaphana*‐sourced isolates

Colony PCR with the universal bacterial primers 27f (5′‐AGA GTT TGA TCM TGG CTC AG‐3′) and 1492r (5′‐TAC GGY TAC CTT GTT ACG ACT T‐3′) (Lane, 1991) was used to generate 16S rRNA gene amplicons from each isolate. Briefly, cells from each pure culture were suspended in 20 µl Milli‐Q water and denatured at 95°C for 10 min. The suspension was then centrifuged at 2000 *g* at 4°C for 2 min, and the supernatant was used as the DNA template for PCR amplification. The PCR was performed with 20 µl Mango Mix™ (Bioline, London, UK), 0.25 µM of each primer and 2 µl of DNA template in a final volume of 40 µl with nuclease free water (Ambion, Thermo Fisher Scientific Inc., Austin, TX, USA). The thermal cycling protocol was as follows: 95°C for 5 min; 35 cycles of 95°C for 1 min, 50°C for 1 min and 72°C for 1 min; and a final extension of 10 min at 72°C. Amplicons were purified and sequenced on an ABI sequencing instrument by Macrogen (Seoul, South Korea) or by the Australian Genome Research Facility using the 1492r primer. Trimmed high‐quality read data from each isolate were used for presumptive identification by querying the 16S rRNA gene sequences via Blastn. For some isolates the near‐complete 16S rRNA gene sequence was determined by sequencing with additional primers [27f, 357f (5′‐CCT ACG GGA GGC AGC AG‐3′ (Muyzer *et al*., 1993)], 926f [5′‐CCG TCA ATT CMT TTR AGT TT‐3′ (Lane *et al*., 1985)], 519r [5′‐GWA TTA CCG CGG CKG CTG‐3′ (Muyzer *et al*., 1993)], 926r [5′‐AAA CTR AAA MGA ATT GAC GG‐3′ (Lane *et al*., 1985), and 1492r]. The six reads for each isolate were aligned using Geneious prime 2019.1.2 (https://www.geneious.com) via the Geneious global alignment tool using default settings and automatic determination of read direction. From this alignment, a consensus sequence for the 16S rRNA gene was constructed based on the frequency of a base and its quality (from chromatogram data) in each alignment column. The consensus sequence length for each of the six isolate pairs ranged from 1352 to 1495 nucleotides. GenBank accession numbers for sequences are shown in Table 1.

### Qualitative free radical scavenging assay

**Fig. 3:**
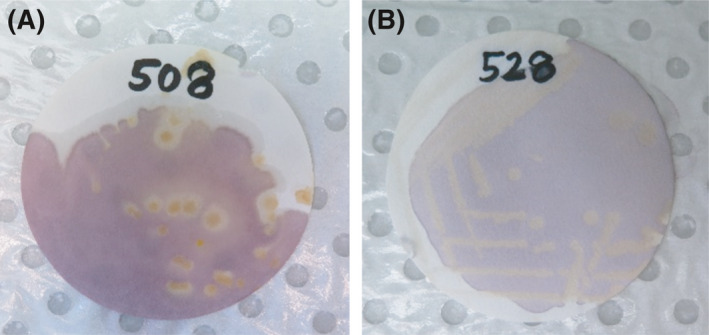
DPPH is a stable free radical that is purple in its oxidized state. When reduced by an antioxidant, a white‐yellow halo will appear around individual bacteria colonies (A), this was qualitatively deemed a positive response. Isolates that did not have a halo around colonies within 3 min of DPPH application were deemed negative (B).

DPPH is a stable free radical that is purple in its oxidized state but becomes white‐yellow if reduced by antioxidants and has been used to identify FRS marine bacteria (Takao *et al*., 1994; Velho‐Pereira *et al*., 2015). To qualitatively assess *E. diaphana*–associated bacterial isolates for FRS ability, a sterile Whatman #1 filter paper was gently pressed against fresh colonies from a streak plate grown for 2‐4 days. Plates (with filter paper) were then incubated overnight at 26°C. The following day, filter papers were removed with forceps and allowed to dry in a fume hood for 30 min, and 500 µl of a 0.2 mM DPPH (Cat# D9132, Sigma‐Aldrich, St. Louis, MO, USA) solution in methanol was applied with a pipette over individual colonies. As a positive control, a few drops of 0.1% (w/v) L‐ascorbic acid (Cat# A7631, Sigma‐Aldrich) were placed on a separate filter. The response of each isolate to DPPH was recorded within 3 min of DPPH application; a positive response was recorded if a white‐yellow halo appeared around individual colonies within 1 min, a weak positive response was assigned to strains that had a halo form between 1 and 3 min after DPPH application, and a negative response was listed for strains that failed to form a halo (Fig. 3). Approximately 700 isolates were screened using the qualitative DPPH assay.

### Quantitative free radical scavenging assay

To quantitatively assess the FRS ability, select isolates were grown in R2A broth (Table S5). Volumes of 50 ml of autoclaved medium were added to sterile 250 ml Erlenmeyer flasks, and each flask was inoculated with an isolate colony grown from a R2A plate culture. Cultures were grown with shaking (150 rpm; Ratek orbital incubator) at 37˚C for 48 h. Sterile uninoculated R2A was used as a control. A minimum of three replicate cultures were grown per isolate. After 48 h, the optical density of each culture was measured at 600 nm (OD_600_, CLARIOstar PLUS, BMG Labtech, Mornington, VIC, Australia), and the cultures (including negative medium controls) were centrifuged at 3000 *g* at 4˚C for 30 min (Allegra X‐12R) to pellet the bacterial cells. Supernatants were collected, frozen at −80˚C, freeze dried (Alpha 1‐4 LDplus, Martin Christ) and stored under inert gas in a dark, dry environment until analysis. Antioxidants were extracted from the freeze‐dried supernatant by resuspending at 50 mg ml^−1^ in 100% methanol, sonicating (Branson 2510) for 5 min and then centrifuging at 3000 *g* at 4˚C for 5 min. Quantitative DPPH assays were run by creating a 1:1 solution of 0.2 mM DPPH in methanol and CFS extract to a final volume of 1 ml, vortexing, and incubation in the dark at room temperature for 30 min. Samples were then vortexed briefly, and three 300 µl replicates for each sample were transferred into a well of a 96‐well plate. Decolorization of DPPH was determined by measuring the decrease in absorbance at 517 nm (Enspire 2300 plate reader, PerkinElmer, Waltham, MA, USA), and the FRS activity was calculated according to the formula, % DPPH scavenging activity = (Control – Sample)/Control × 100, where Control is the absorbance of the DPPH control (1:1 0.2 mM DPPH:methanol) and Sample is the absorbance of CFS extract in DPPH. All samples were measured against a 100% methanol blank. Positive controls consisting of 0.01–0.001% (w/v) L‐ascorbic acid were run on each 96‐well plate. FRS activity ranged from 0–90%.

### Catalase assay

The pelleted cells from above were resuspended in 4.5 ml fRSS and 500 µl 3% (w/v) H_2_O_2_ (Rosado *et al*., 2018). If bubbles appeared, the organism was considered catalase positive. If there were no bubbles, the organism was classified as catalase negative.

### Inhibition testing

Each paired set of high and low FRS strains were inoculated crosswise along the middle of R2A plates to test for antagonism. Plates were kept at 26°C and monitored daily for up to 7 days for antagonistic activity by documenting the presence or absence of both inoculated isolates and if there was a zone of inhibition between them.

### Bacterial growth curves

Diluted cultures for each isolate were established by resuspending one isolate colony grown from a R2A plate culture in 500 µl of autoclaved R2A broth (Table S5). Volumes of 2 ml of autoclaved R2A broth were then added to each well of two sterile 24‐well plates. Three replicate randomly distributed wells were inoculated with 100 µl of the respective diluted cultures. There were six replicates of sterile uninoculated R2A blanks per plate. Bacterial growth curves were documented for each of the twelve selected isolates at 37°C over 56 h. Mixing was set to 300 rpm for the duration of the growth curve and OD_600_ measurements were taken every 10 min (CLARIOstar PLUS, BMG Labtech, Mornington, VIC, Australia). For each isolate, the growth curves for the triplicate cultures were averaged and blank corrected. Results were plotted in the MARS Data Analysis Software with OD_600_ values shown over time.

### Phylogenetic analysis

All partial 16S rRNA gene sequences (842) were aligned with reference sequences (72) of closely related organisms using Geneious prime 2019.1.2 (https://www.geneious.com). This alignment was used to construct a neighbor‐joining phylogenetic tree using the Jukes‐Cantor method. Maximum‐likelihood dendrograms were generated with bootstrap values of 1000.

### Whole genome sequence analysis

High FRS isolates along with conspecific or congeneric low FRS isolates were selected for genome sequencing; in total, six pairs of isolates were sequenced. Genomic DNA was isolated from a single colony using a JANUS Chemagic Workstation and Chemagic Viral DNA/RNA kit (PerkinElmer). Libraries were prepared with the Nextera XT DNA sample preparation kit (Illumina, San Diego, CA, USA). Readsets were produced using the Illumina sequencing platform (Instrument: Illumina NextSeq 500, 150 base, paired‐end) and the whole genome shotgun (WGS) method. Read depth coverage was approximately 100 times assuming a genome size of 4 M bases.

Illumina readsets for each isolate were assembled using Skesa (Souvorov *et al*., 2018), and the draft genome sequence was annotated using Prokka (Seemann, 2014). A genome sequence–based taxonomic classification for each isolate was determined using Kraken2 (Wood and Salzberg, 2014) with the Genome Taxonomy Database (GTDB; Parks *et al*., 2020) as the curated genomic data source. Classification was primarily based on the genome sequence of related isolates (within the relevant species where possible), which were obtained from GenBank. In situations where genomes of taxonomically relevant strains were available, a species‐level classification was possible. Where available, closed genome sequences from GenBank were used for comparative genomics analysis. For each of the six pairs of isolates, core genome (i.e. genes shared between the isolate pair) comparisons were performed, as implemented in Nullarbor (https://github.com/tseemann/nullarbor), with phylogenies inferred using core SNP differences. Genes for DMSP synthesis and degradation, vitamin B_12_ synthesis, and catalase were identified from the annotated genome sequence (GFF format) produced by Prokka; specific genes were identified by both name and Refseq accession number.

### 16S rRNA gene copy number estimation

The 16S rRNA gene copy number of the 12 draft genomes was predicted by the 16Stimator pipeline (Perisin *et al*., 2016). Briefly, all the 12 genomic assemblies were submitted to the rapid annotations using the subsystems technology (RAST) server (Brettin *et al*., 2015), and the positions of 16S rRNA and a set of single‐copied housekeeping genes (Table S6) were extracted from the RAST annotations. The clean read sets were mapped back to the corresponding genomic assemblies by Bowtie 2 (Langmead and Salzberg, 2012) to determine the read depth of each position. Finally, the 16S copy number of each isolate was calculated by dividing the median depth of 16S gene by the median depth of the single‐copied housekeeping genes after the read depths were calibrated by the model parameters provided by 16Stimator.

### Statistical analysis

CFU counts were analyzed in R (v3.6.2, R Core Team, 2019) by first checking the assumptions of equal variance and homogeneity. An analysis of variance test was used to detect differences in the mean number of bacterial colonies from each anemone genotype by solid growth media (R2A or MA). A one‐way analysis of variance (one‐way ANOVA; Chambers and Hastie, 1992) was used to determine if there were significant differences between FRS abilities of selected high FRS, low FRS, and media controls, and pairwise comparisons were performed using Tukey’s HSD (Miller, 1981; Yandell, 2017). Each congeneric/conspecific pair and media control was tested to determine if data met the assumptions of normality and homoscedasticity. If either assumption was violated, the non‐parametic Kruskal‐Wallis rank sum test (Holland and Wolfe, 1973) was used with a Dunn test (Dunn, 1964) for multiple comparisons [*P*‐values adjusted with the Benjamini‐Hochberg method (Ferreira and Zwinderman, 2006)] with the R package “FSA” (Ogle, 2017).

## Conflict of interest

The authors declare that they have no conflict of interest.

## Author contributions

AMD, MvO and LLB conceived and designed the study. AMD performed the sampling and sample processing. AMD, DB and HL completed bioinformatic analyses. AMD wrote the first draft. All authors edited and approved the final manuscript.

## Supporting information

**Fig. S1.** Growth curve of high FRS bacterial isolate MMSF00046 (*Winogradskyella poriferorum*) over 56 h at 300 rpm and 37°C.
**Fig. S2.** Growth curve of low FRS bacterial isolate MMSF00910 (*Winogradskyella poriferorum*) over 56 h at 300 rpm and 37°C.
**Fig. S3.** Growth curve of high FRS bacterial isolate MMSF00068 (*Micococcus luteus*) over 56 h at 300 rpm and 37°C.
**Fig. S4.** Growth curve of low FRS bacterial isolate MMSF00107 (*Micococcus* *yunnanensis*) over 56 h at 300 rpm and 37°C.
**Fig. S5.** Growth curve of high FRS bacterial isolate MMSF00132 (*Labrenzia aggregata*) over 56 h at 300 rpm and 37°C.
**Fig. S6.** Growth curve of low FRS bacterial isolate MMSF00249 (*Labrenzia aggregata*) over 56 h at 300 rpm and 37°C.
**Fig. S7.** Growth curve of high FRS bacterial isolate MMSF00958 (*Alteromonas macleodii*) over 56 h at 300 rpm and 37°C.
**Fig. S8.** Growth curve of low FRS bacterial isolate MMSF00257 (*Alteromonas macleodii*) over 56 h at 300 rpm and 37°C.
**Fig. S9.** Growth curve of high FRS bacterial isolate MMSF01163 (*Alteromonas oceani*) over 56 h at 300 rpm and 37°C.
**Fig. S10.** Growth curve of low FRS bacterial isolate MMSF00404 (*Alteromonas oceani*) over 56 h at 300 rpm and 37°C.
**Fig. S11.** Growth curve of high FRS bacterial isolate MMSF01190 (*Marinobacter salsuginis*) over 56 h at 300 rpm and 37°C.
**Fig. S12.** Growth curve of low FRS bacterial isolate MMSF00964 (*Marinobacter salsuginis*) over 56 h at 300 rpm and 37°C.

**Table S1.** Isolate Genome Sequence Data Summary. Strains are presented as high FRS (grey) followed by low FRS (white). 16S rRNA gene presumptive identity is derived from the NCBI classification of near‐complete 16S rRNA gene sequences. *We were unable to determine the 16S rRNA copy number of isolate MMSF00068.
**Table S2.** Pairwise comparison of the genome sequences between the pairs of isolates.
**Table S3.** Search outcomes for genes of interest.
**Table S4.** Summary of vitamin B_12_ biosynthesis pathway genes. A “+” indicates the presence of the gene in the respective isolate, whereas a “–” represents the absence of that gene. Genes in red were not found in any isolate.
**Table S5.** Composition of R2A broth adjusted to suit marine bacteria. Final pH = 7.2 +/‐ 0.2 at 26 °C. R2A broth was made by suspending 43.12 g of combined reagents in 1 l of MilliQ water, dissolving the medium completely, and sterilization by autoclaving at 121˚C for 15 min.
**Table S6.** Single‐copy housekeeping genes extracted from the RAST annotations.

## Data Availability

WGS raw reads are freely available in the Sequence Read Archive under BioProject PRJNA574193; the complete data set is listed in Table S1.
