# Supplementary material for: Development of a free radical scavenging bacterial consortium to mitigate oxidative stress in cnidarians
Source: Microb Biotechnol. 2021 Jul 14;14(5):2025–40. doi: 10.1111/1751-7915.13877 (PMC8449677; doi:10.1111/1751-7915.13877)
Supplement: Supplementary file 1 — Fig. S1. Growth curve of high FRS bacterial isolate MMSF00046 (Winogradskyella poriferorum) over 56 h at 300 rpm and 37°C. Fig. S2. Growth curve of low FRS bacterial isolate MMSF00910 (Winogradskyella poriferorum) over 56 h at 300 rpm and 37°C. Fig. S3. Growth curve of high FRS bacterial isolate MMSF00068 (Micococcus luteus) over 56 h at 300 rpm and 37°C. Fig. S4. Growth curve of low FRS bacterial isolate MMSF00107 (Micococcus yunnanensis) over 56 h at 300 rpm and 37°C. Fig. S5. Growth curve of high FRS bacterial isolate MMSF00132 (Labrenzia aggregata) over 56 h at 300 rpm and 37°C. Fig. S6. Growth curve of low FRS bacterial isolate MMSF00249 (Labrenzia aggregata) over 56 h at 300 rpm and 37°C. Fig. S7. Growth curve of high FRS bacterial isolate MMSF00958 (Alteromonas macleodii) over 56 h at 300 rpm and 37°C. Fig. S8. Growth curve of low FRS bacterial isolate MMSF00257 (Alteromonas macleodii) over 56 h at 300 rpm and 37°C. Fig. S9. Growth curve of high FRS bacterial isolate MMSF01163 (Alteromonas oceani) over 56 h at 300 rpm and 37°C. Fig. S10. Growth curve of low FRS bacterial isolate MMSF00404 (Alteromonas oceani) over 56 h at 300 rpm and 37°C. Fig. S11. Growth curve of high FRS bacterial isolate MMSF01190 (Marinobacter salsuginis) over 56 h at 300 rpm and 37°C. Fig. S12. Growth curve of low FRS bacterial isolate MMSF00964 (Marinobacter salsuginis) over 56 h at 300 rpm and 37°C. [file MBT2-14-2025-s001.docx]

**Figure S1:** Growth curve of high FRS bacterial isolate MMSF00046 (Winogradskyella poriferorum) over 56 h at 300 rpm and 37°C.


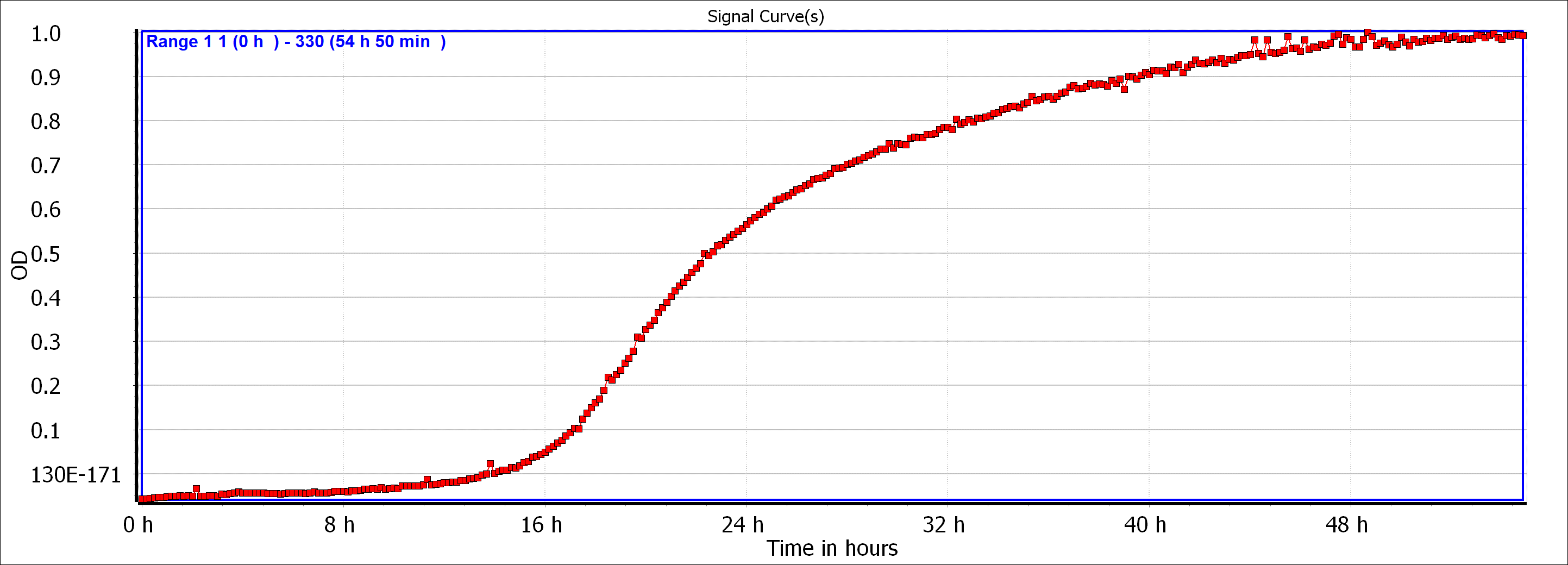


**Figure S2:** Growth curve of low FRS bacterial isolate MMSF00910 (Winogradskyella poriferorum) over 56 h at 300 rpm and 37°C.


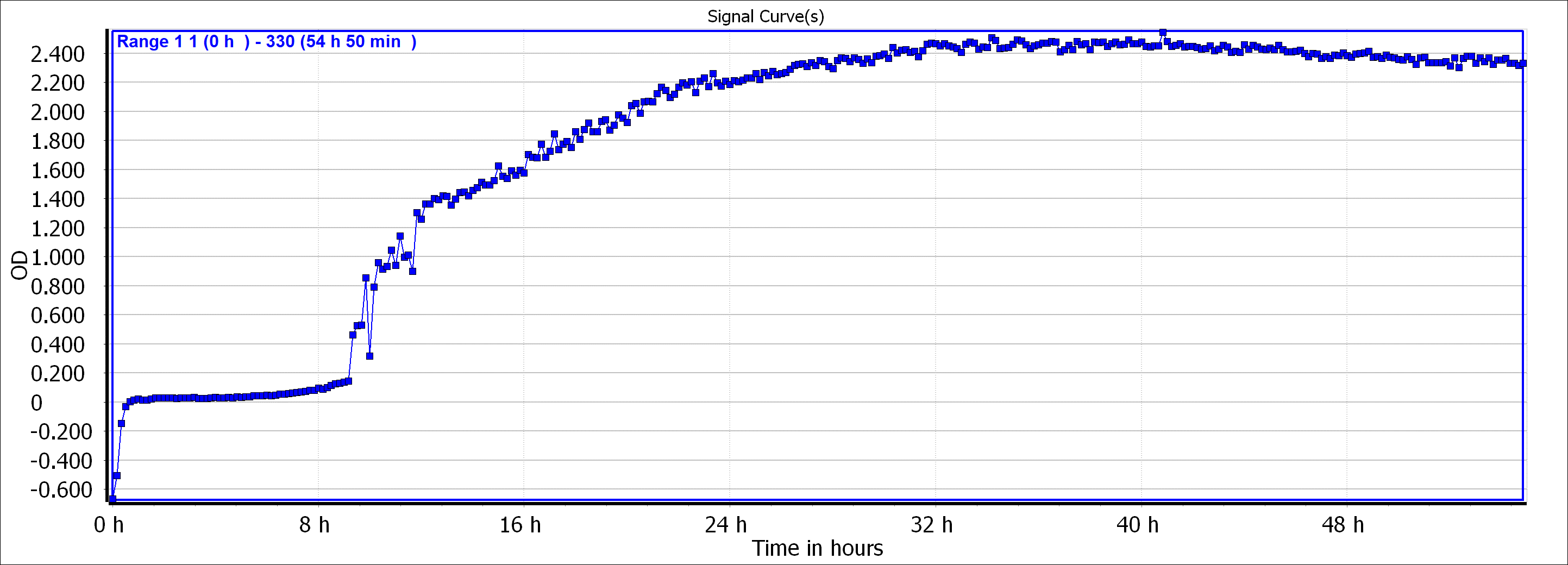


**Figure S3:** Growth curve of high FRS bacterial isolate MMSF00068 (Micococcus luteus) over 56 h at 300 rpm and 37°C.


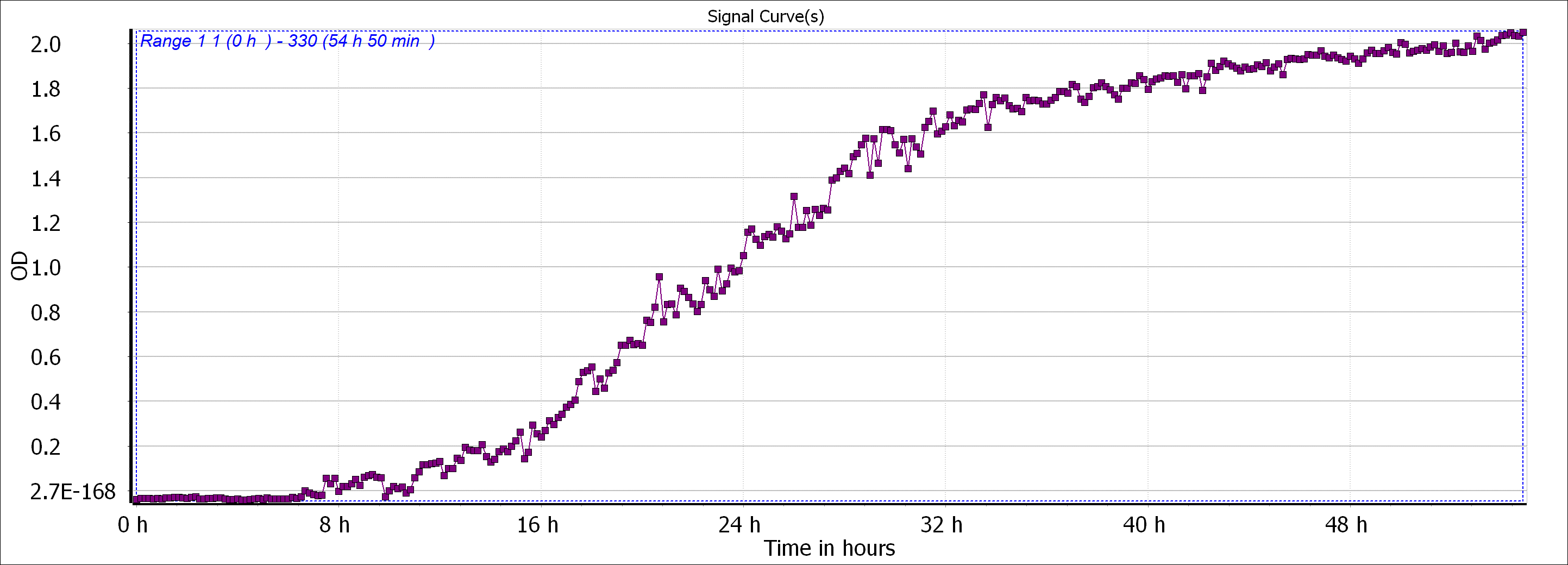


**Figure S4:** Growth curve of low FRS bacterial isolate MMSF00107 (Micococcus yunnanensis) over 56 h at 300 rpm and 37°C.


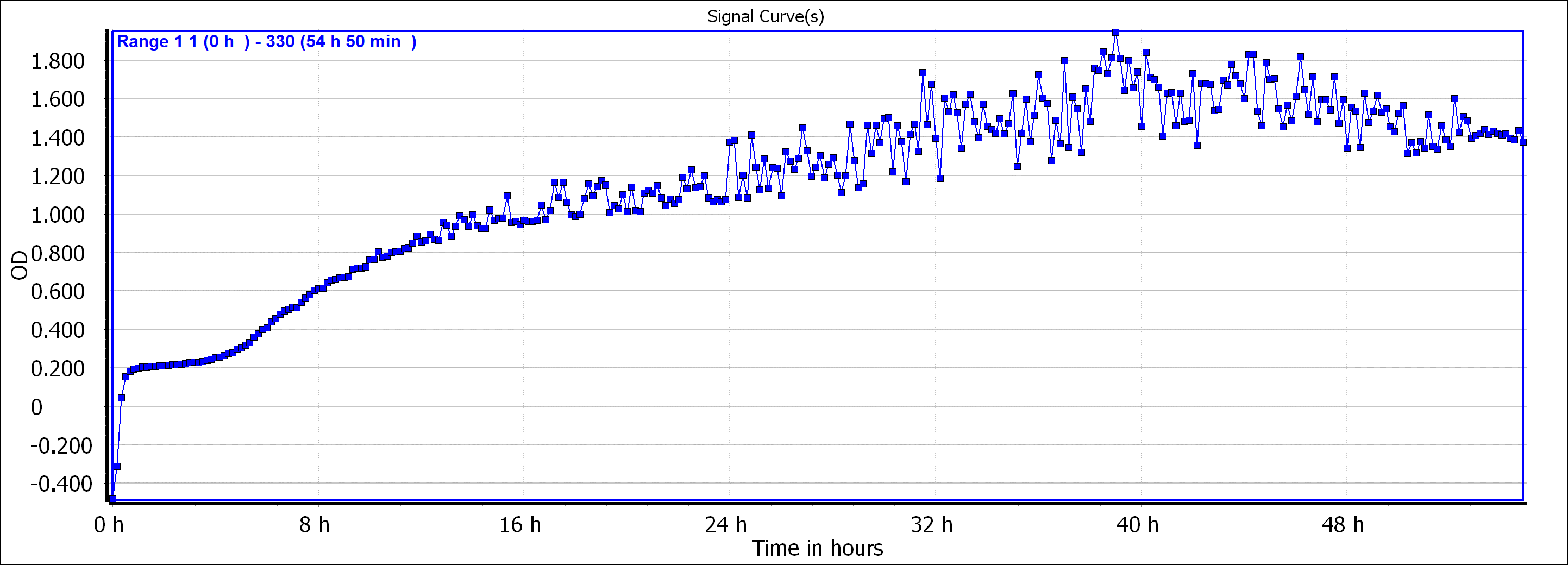


**Figure S5:** Growth curve of high FRS bacterial isolate MMSF00132 (Labrenzia aggregata) over 56 h at 300 rpm and 37°C.


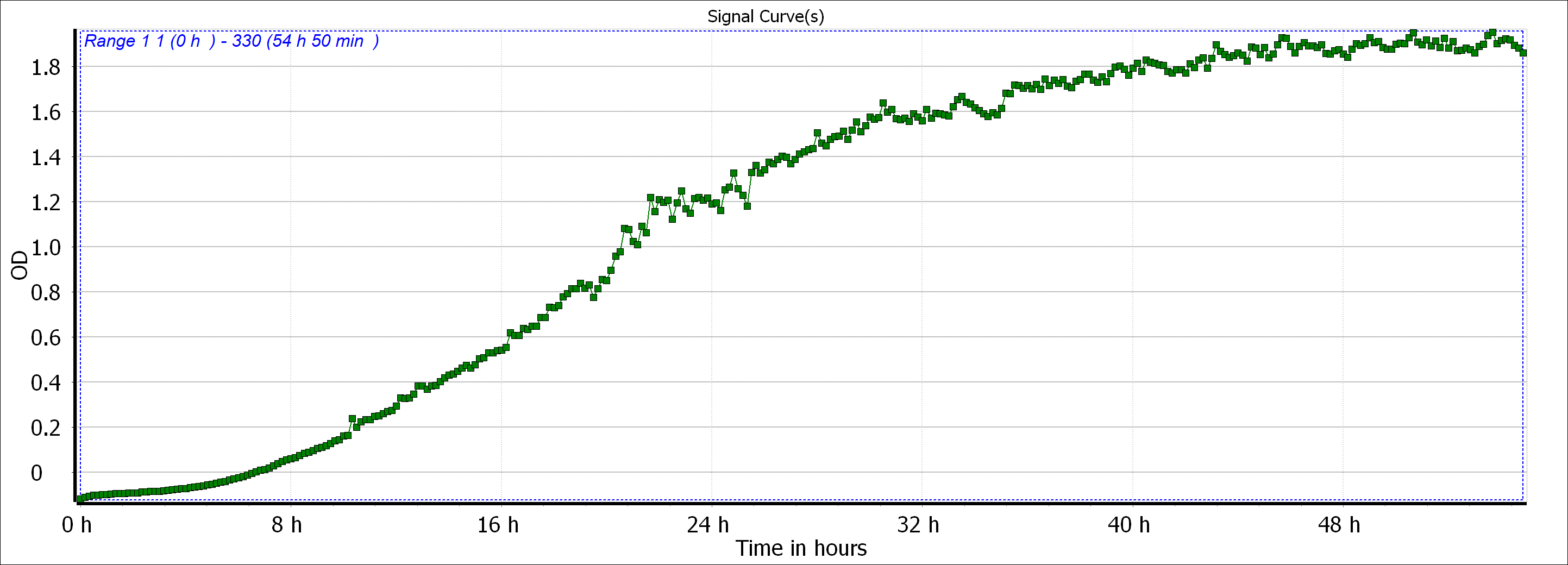


**Figure S6:** Growth curve of low FRS bacterial isolate MMSF00249 (Labrenzia aggregata) over 56 h at 300 rpm and 37°C.


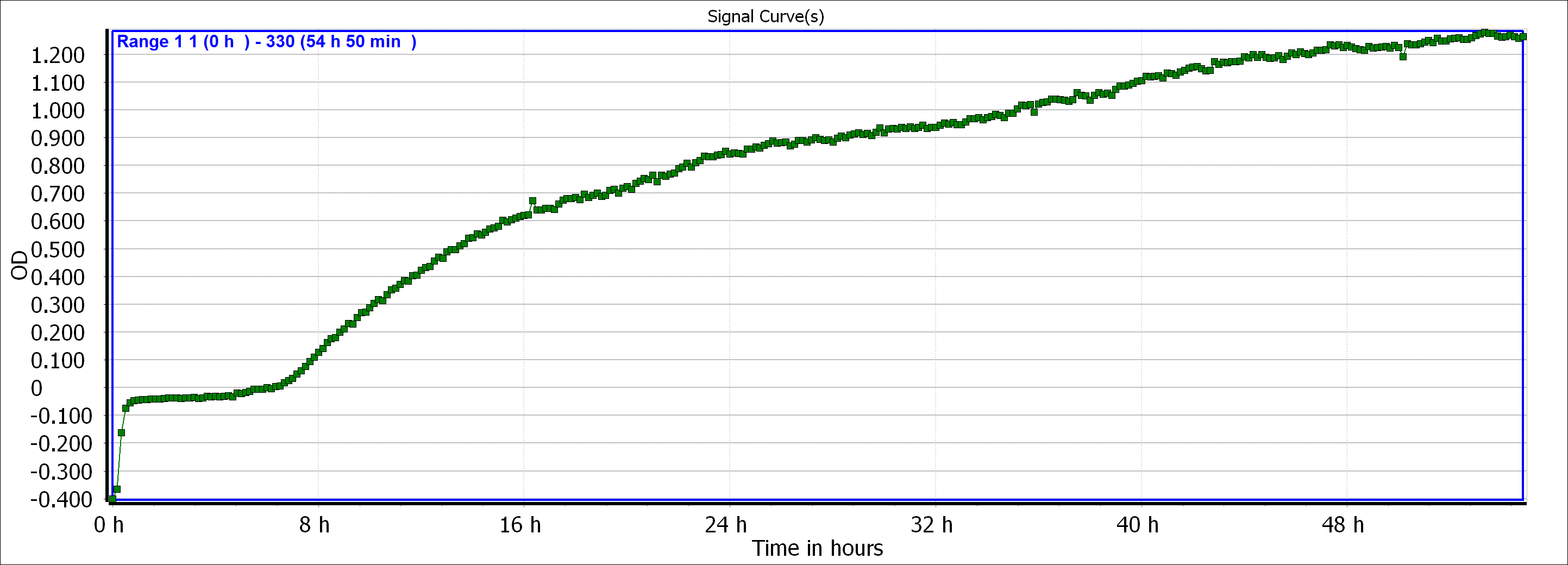


**Figure S7:** Growth curve of high FRS bacterial isolate MMSF00958 (Alteromonas macleodii) over 56 h at 300 rpm and 37°C.


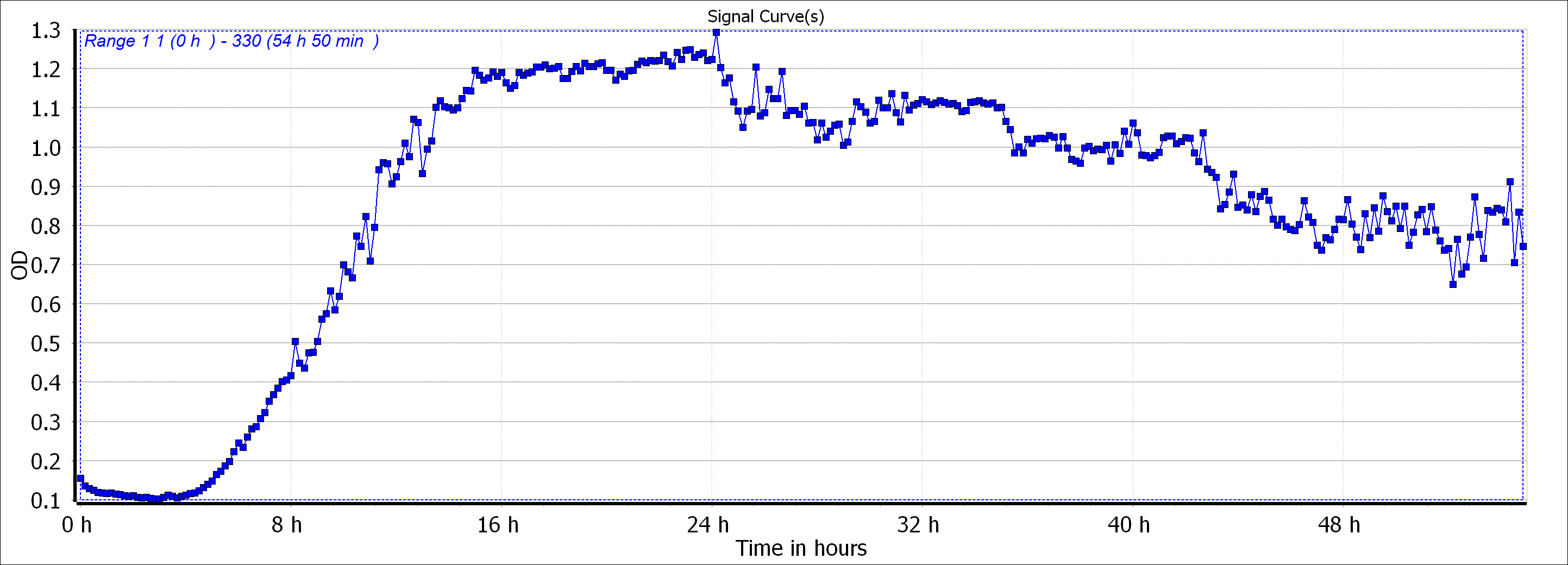


**Figure S8** Growth curve of low FRS bacterial isolate MMSF00257 (Alteromonas macleodii) over 56 h at 300 rpm and 37°C.


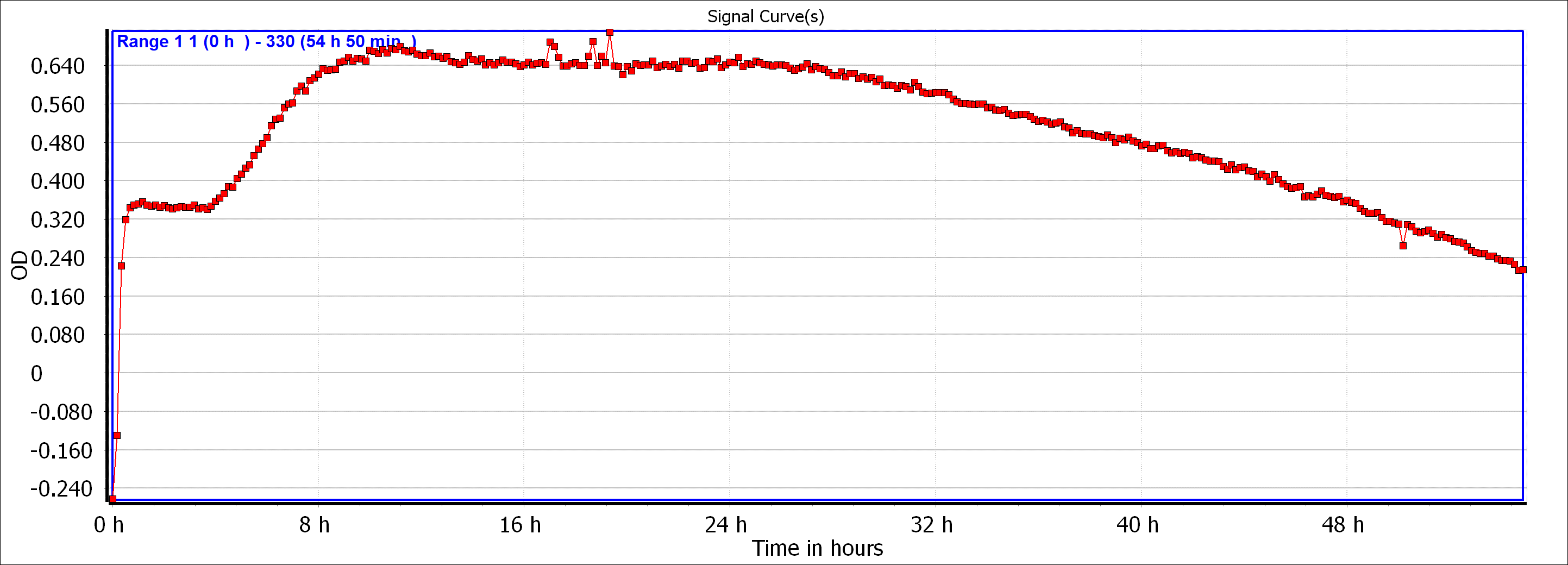


**Figure S9:** Growth curve of high FRS bacterial isolate MMSF01163 (Alteromonas oceani) over 56 h at 300 rpm and 37°C.


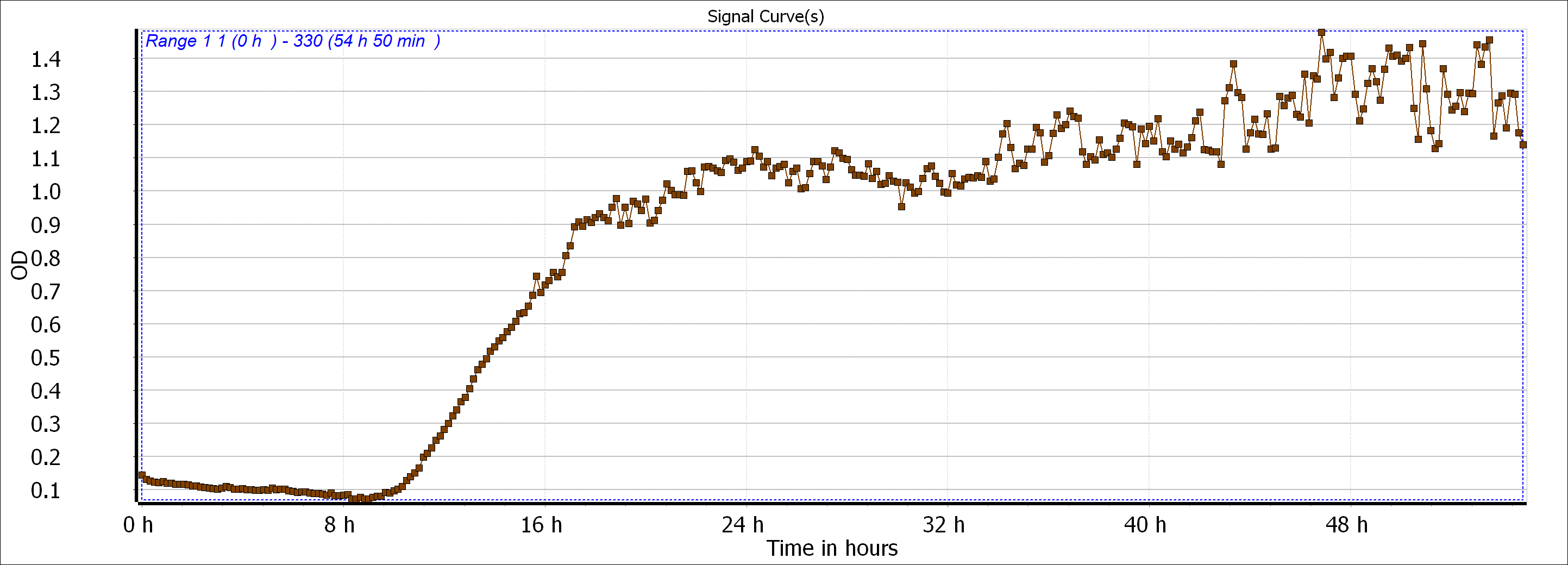


**Figure S10:** Growth curve of low FRS bacterial isolate MMSF00404 (Alteromonas oceani) over 56 h at 300 rpm and 37°C.


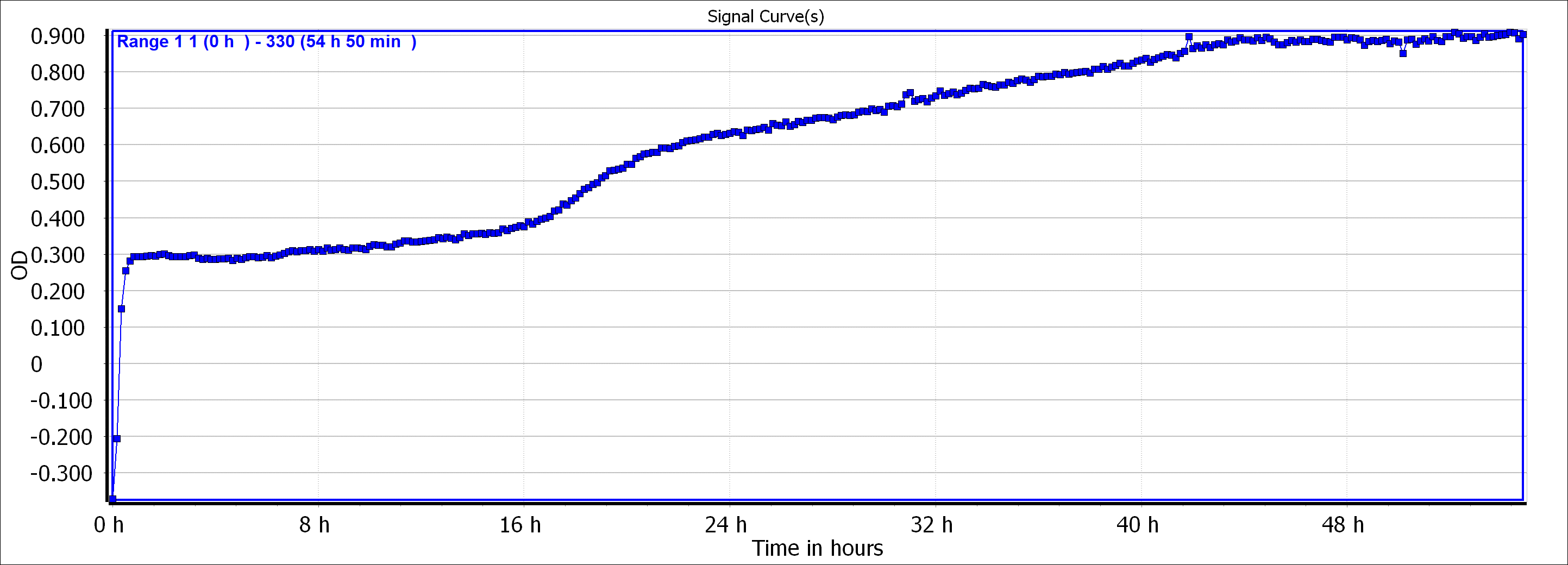


**Figure S11:** Growth curve of high FRS bacterial isolate MMSF01190 (Marinobacter salsuginis) over 56 h at 300 rpm and 37°C.


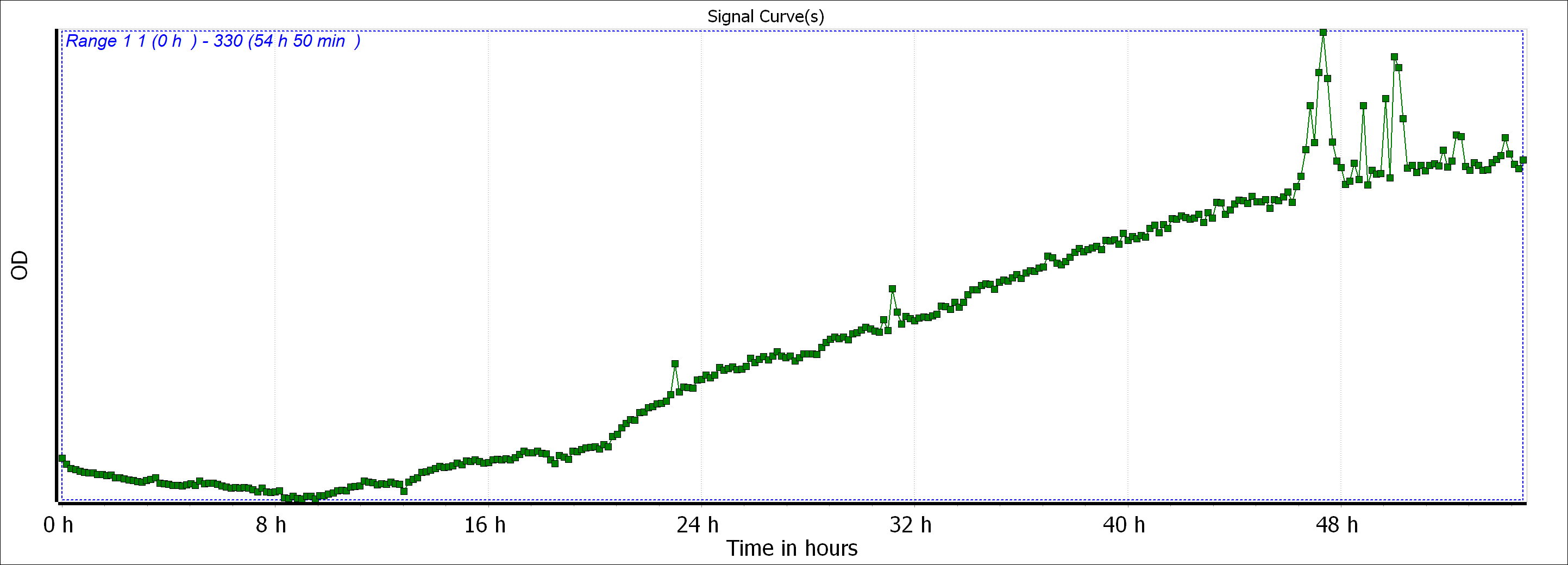


**Figure S12:** Growth curve of low FRS bacterial isolate MMSF00964 (Marinobacter salsuginis) over 56 h at 300 rpm and 37°C.
