# Supplementary material for: Development of a free radical scavenging bacterial consortium to mitigate oxidative stress in cnidarians
Source: Microb Biotechnol. 2021 Jul 14;14(5):2025–40. doi: 10.1111/1751-7915.13877 (PMC8449677; doi:10.1111/1751-7915.13877)
Supplement: Supplementary file 2 — Table S1. Isolate Genome Sequence Data Summary. Strains are presented as high FRS (grey) followed by low FRS (white). 16S rRNA gene presumptive identity is derived from the NCBI classification of near‐complete 16S rRNA gene sequences. *We were unable to determine the 16S rRNA copy number of isolate MMSF00068. Table S2. Pairwise comparison of the genome sequences between the pairs of isolates. Table S3. Search outcomes for genes of interest. Table S4. Summary of vitamin B12 biosynthesis pathway genes. A “+” indicates the presence of the gene in the respective isolate, whereas a “–” represents the absence of that gene. Genes in red were not found in any isolate. Table S5. Composition of R2A broth adjusted to suit marine bacteria. Final pH = 7.2 +/‐ 0.2 at 26 °C. R2A broth was made by suspending 43.12 g of combined reagents in 1 l of MilliQ water, dissolving the medium completely, and sterilization by autoclaving at 121˚C for 15 min. Table S6. Single‐copy housekeeping genes extracted from the RAST annotations. [file MBT2-14-2025-s002.docx]

**Table S1**: Isolate Genome Sequence Data Summary. Strains are presented as high FRS (grey) followed by low FRS (white). 16S rRNA gene presumptive identity is derived from the NCBI classification of near-complete 16S rRNA gene sequences. *We were unable to determine the 16S rRNA copy number of isolate MMSF00068.

| **Isolate** | **Genus Level Classification** | **Genus % Confidence** | **Family** | **Family % Confidence** | **SRA Accession** | **Sample Accession** |
| --- | --- | --- | --- | --- | --- | --- |
| **MMSF00257** | *Alteromonas* | 96.65 | *Alteromonadaceae* | 96.74 | SRR10186803 | SAMN12851724 |
| **MMSF00958** | *Alteromonas* | 92.93 | *Alteromonadaceae* | 92.98 | SRR10186806 | SAMN12851731 |
| **MMSF01163** | *Alteromonas* | 9.28 | *Alteromonadaceae* | 12.01 | SRR10186808 | SAMN12851721 |
| **MMSF00404** | *Alteromonas* | 8.15 | *Alteromonadaceae* | 10.49 | SRR10186805 | SAMN12851732 |
| **MMSF00132** | *Labrenzia* | 90.9 | *Rhodobacteraceae* | 90.91 | SRR10186800 | SAMN12851727 |
| **MMSF00249** | *Labrenzia* | 90.83 | *Rhodobacteraceae* | 90.83 | SRR10186799 | SAMN12851728 |
| **MMSF00964** | *Marinobacter* | 59.99 | *Alteromonadaceae* | 60.1 | SRR10186802 | SAMN12851725 |
| **MMSF01190** | *Marinobacter* | 65.77 | *Alteromonadaceae* | 65.85 | SRR10186797 | SAMN12851730 |
| **MMSF00068** | *Micrococcus* | 71.81 | *Micrococcaceae* | 72.88 | SRR10186807 | SAMN12851722 |
| **MMSF00107** | *Micrococcus* | 83.18 | *Micrococcaceae* | 84.87 | SRR10186798 | SAMN12851729 |
| **MMSF00046** | *Winogradskyella* | 49.63 | *Flavobacteriaceae* | 49.72 | SRR10186801 | SAMN12851726 |
| **MMSF00910** | *Winogradskyella* | 54.44 | *Flavobacteriaceae* | 54.53 | SRR10186804 | SAMN12851723 |

| **Isolate** | **16S rRNA gene presumptive identity** | **Confidence** | **Reads** | **Total Bases** | **G+C%** | **Avg. Read Length** | **Max Read Length** | **Avg Quality** |
| --- | --- | --- | --- | --- | --- | --- | --- | --- |
| **MMSF00257** | *Alteromonas macleodii* | 1.00 | 2233080 | 334079592 | 44.7 | 149 | 151 | 30.9 |
| **MMSF00958** | *Alteromonas macleodii* | 1.00 | 2971718 | 443870758 | 44.6 | 149 | 151 | 31.9 |
| **MMSF01163** | *Alteromonas oceani* | 0.83 | 2757340 | 412741366 | 48.7 | 149 | 151 | 33.7 |
| **MMSF00404** | *Alteromonas oceani* | 0.83 | 2067800 | 306005114 | 48.7 | 147 | 151 | 33.3 |
| **MMSF00132** | *Labrenzia aggregata* | 0.98 | 3758944 | 557529936 | 59.2 | 148 | 151 | 31.1 |
| **MMSF00249** | *Labrenzia aggregata* | 0.98 | 3333098 | 497032699 | 59.3 | 149 | 151 | 30.7 |
| **MMSF00964** | *Marinobacter salsuginis* | 1.00 | 1713198 | 256635737 | 57.1 | 149 | 151 | 31 |
| **MMSF01190** | *Marinobacter salsuginis* | 1.00 | 3239568 | 483874048 | 57.1 | 149 | 151 | 33.1 |
| **MMSF00068** | *Micrococcus luteus* | 0.99 | 2658898 | 398496031 | 72.4 | 149 | 151 | 30.3 |
| **MMSF00107** | *Micrococcus yunnanensis* | 0.99 | 2323654 | 348617190 | 72.8 | 150 | 151 | 31.9 |
| **MMSF00046** | *Winogradskyella poriferorum* | 0.59 | 1930584 | 285978916 | 35 | 148 | 151 | 31.8 |
| **MMSF00910** | *Winogradskyella poriferorum* | 0.59 | 3939880 | 579510587 | 35.6 | 147 | 151 | 33.9 |

| **Isolate** | **Est. Read Coverage** | **Contigs in Draft Genome** | **Bases in Draft Genome** | **Min Contig** | **Avg Contig** | **Max Contig** | **N50** | **16S rRNA gene copy number** |
| --- | --- | --- | --- | --- | --- | --- | --- | --- |
| **MMSF00257** | 69 | 136 | 4831263 | 515 | 35523 | 210048 | 72973 | 5.15 |
| **MMSF00958** | 94 | 42 | 4732026 | 620 | 112667 | 580903 | 303003 | 4.79 |
| **MMSF01163** | 75 | 100 | 5507488 | 526 | 55074 | 394030 | 155957 | 4.18 |
| **MMSF00404** | 51 | 111 | 6014142 | 502 | 54181 | 256548 | 93725 | 3.97 |
| **MMSF00132** | 82 | 34 | 6792087 | 855 | 199767 | 1019681 | 302139 | 3.57 |
| **MMSF00249** | 73 | 44 | 6791310 | 851 | 154347 | 1286212 | 294458 | 3.02 |
| **MMSF00964** | 56 | 41 | 4588310 | 512 | 111910 | 932206 | 291012 | 3.67 |
| **MMSF01190** | 110 | 40 | 4404819 | 529 | 110120 | 769441 | 404351 | 3.21 |
| **MMSF00068** | 160 | 316 | 2484978 | 509 | 7863 | 78799 | 12670 | ***** |
| **MMSF00107** | 143 | 501 | 2435904 | 515 | 4862 | 42365 | 7717 | 1.75 |
| **MMSF00046** | 83 | 104 | 3446386 | 553 | 33138 | 299826 | 78549 | 1.03 |
| **MMSF00910** | 168 | 50 | 3456468 | 553 | 69129 | 355406 | 162623 | 0.77 |

**Table S2**: Pairwise comparison of the genome sequences between the pairs of isolates

| **NCBI Classification** | **Type strain** | **Core Genome** | **SNP difference** |
| --- | --- | --- | --- |
| *Alteromonas macleodii* | ATCC 27126 | 85% | ~60000 |
| *Alteromonas oceani* | S35 | 60% | ~190000 |
| *Labrenzia aggregata* | IAM12614 | 55% | 5 |
| *Marinobacter salsuginis* | SD14B | 15% | ~120000 |
| *Micrococcus* spp. | NCTC2665 | 70% | ~35000 |
| *Winogradskyella poriferorum* | NA | NA | 10 |

**Table S3:** Search outcomes for genes of interest.

| Isolate | NCBI Classification | Gene | Gene Classification | Contig | Start | End | Strand |
| --- | --- | --- | --- | --- | --- | --- | --- |
| MMSF00132 | *Labrenzia aggregata* | *dddL* | positive | Contig_16_56.34 | 104067 | 104756 | - |
| MMSF00249 | *Labrenzia aggregata* | *dddL* | positive | Contig_18_45.1167 | 104045 | 104734 | - |
| MMSF00132 | *Labrenzia aggregata* | *dddP* | positive x2 (Copy 1) | Contig_6_57.2063 | 50787 | 52046 | + |
| MMSF00132 | *Labrenzia aggregata* | *dddP* | positive x2 (Copy 2) | Contig_6_57.2063 | 650583 | 651881 | - |
| MMSF00249 | *Labrenzia aggregata* | *dddP* | positive x2 (Copy 1) | Contig_17_46.7174 | 317316 | 318575 | + |
| MMSF00249 | *Labrenzia aggregata* | *dddP* | positive x2 (Copy 2) | Contig_17_46.7174 | 917112 | 918410 | - |
| MMSF00132 | *Labrenzia aggregata* | *dsyB* | positive | Contig_32_56.2994 | 210397 | 211419 | + |
| MMSF00249 | *Labrenzia aggregata* | *dsyB* | positive | Contig_4_46.0396 | 460215 | 461237 | + |
| MMSF00046 | *Winogradskyella poriferorum* | *katG* | positive | Contig_15_55.0851 | 16430 | 18661 | + |
| MMSF00068 | *Micrococcus luteus* | *katG* | ND; katA & katE detected | NA | NA | NA | NA |
| MMSF00107 | *Micrococcus yunnanensis* | *katG* | ND; katA & katE detected | NA | NA | NA | NA |
| MMSF00132 | *Labrenzia aggregata* | *katG* | positive | Contig_6_57.2063 | 425873 | 428044 | - |
| MMSF00249 | *Labrenzia aggregata* | *katG* | positive | Contig_17_46.7174 | 692402 | 694573 | - |
| MMSF00257 | *Alteromonas macleodii* | *katG* | positive | Contig_101_46.6864 | 22130 | 24502 | + |
| MMSF00404 | *Alteromonas oceani* | *katG* | positive x2 (Copy 1) | Contig_26_39.8992 | 70873 | 73035 | - |
| MMSF00404 | *Alteromonas oceani* | *katG* | positive x2 (Copy 2) | Contig_52_41.1626 | 55994 | 58225 | - |
| MMSF00910 | *Winogradskyella poriferorum* | *katG* | positive | Contig_39_101.483 | 25621 | 27852 | + |
| MMSF00958 | *Alteromonas macleodii* | *katG* | positive | Contig_21_66.072 | 22086 | 24458 | + |
| MMSF00964 | *Marinobacter salsuginis* | *katG* | positive | Contig_25_36.8412 | 50391 | 52565 | - |
| MMSF01163 | *Alteromonas oceani* | *katG* | positive x2 (Copy 1) | Contig_46_63.2119 | 54248 | 56479 | - |
| MMSF01163 | *Alteromonas oceani* | *katG* | positive x2 (Copy 2) | Contig_86_59.9301 | 369720 | 371882 | - |
| MMSF01190 | *Marinobacter salsuginis* | *katG* | positive | Contig_22_84.8909 | 7780 | 9954 | + |
| MMSF00132 | *Labrenzia aggregata* | *cobA* | positive | Contig_6_57.2063 | 552920 | 553741 | - |
| MMSF00249 | *Labrenzia aggregata* | *cobA* | positive | Contig_17_46.7174 | 819449 | 820270 | - |
| MMSF00132 | *Labrenzia aggregata* | *cobB* | positive | Contig_6_57.2063 | 551611 | 552930 | - |
| MMSF00249 | *Labrenzia aggregata* | *cobB* | positive | Contig_17_46.7174 | 818140 | 819459 | - |
| MMSF00132 | *Labrenzia aggregata* | *cobC* | positive | Contig_6_57.2063 | 842748 | 843392 | - |
| MMSF00249 | *Labrenzia aggregata* | *cobC* | positive | Contig_17_46.7174 | 1109277 | 1E+06 | - |
| MMSF00132 | *Labrenzia aggregata* | *cobD* | positive x2 | Contig_26_56.6932 | 208207 | 209184 | - |
| MMSF00132 | *Labrenzia aggregata* | *cobD* | positive x2 | Contig_26_56.6932 | 209194 | 210195 | - |
| MMSF00249 | *Labrenzia aggregata* | *cobD* | positive x2 | Contig_15_47.504 | 84229 | 85230 | + |
| MMSF00249 | *Labrenzia aggregata* | *cobD* | positive x2 | Contig_15_47.504 | 85240 | 86217 | + |
| MMSF00132 | *Labrenzia aggregata* | *cobH* | positive | Contig_6_57.2063 | 545294 | 545950 | + |
| MMSF00249 | *Labrenzia aggregata* | *cobH* | positive | Contig_17_46.7174 | 811823 | 812479 | + |
| MMSF00132 | *Labrenzia aggregata* | *cobI* | positive | Contig_6_57.2063 | 547369 | 548139 | + |
| MMSF00249 | *Labrenzia aggregata* | *cobI* | positive | Contig_17_46.7174 | 813898 | 814668 | + |
| MMSF00132 | *Labrenzia aggregata* | *cobK* | positive | Contig_6_57.2063 | 555025 | 555786 | + |
| MMSF00249 | *Labrenzia aggregata* | *cobK* | positive | Contig_17_46.7174 | 821554 | 822315 | + |
| MMSF00132 | *Labrenzia aggregata* | *cobL* | positive | Contig_6_57.2063 | 546203 | 547417 | + |
| MMSF00249 | *Labrenzia aggregata* | *cobL* | positive | Contig_17_46.7174 | 812732 | 813946 | + |
| MMSF00132 | *Labrenzia aggregata* | *cobM* | positive | Contig_6_57.2063 | 550028 | 550801 | + |
| MMSF00249 | *Labrenzia aggregata* | *cobM* | positive | Contig_17_46.7174 | 816557 | 817330 | + |
| MMSF00132 | *Labrenzia aggregata* | *cobN* | positive | Contig_26_56.6932 | 201965 | 205723 | + |
| MMSF00249 | *Labrenzia aggregata* | *cobN* | positive | Contig_15_47.504 | 88701 | 92459 | - |
| MMSF00132 | *Labrenzia aggregata* | *cobO* | positive | Contig_26_56.6932 | 205720 | 206358 | + |
| MMSF00249 | *Labrenzia aggregata* | *cobO* | positive | Contig_15_47.504 | 88066 | 88704 | - |
| MMSF00964 | *Marinobacter salsuginis* | *cobO* | positive | Contig_25_36.8412 | 168373 | 168930 | + |
| MMSF01190 | *Marinobacter salsuginis* | *cobO* | positive | Contig_21_84.3861 | 85977 | 86534 | + |
| MMSF00132 | *Labrenzia aggregata* | *cobP* | positive | Contig_26_56.6932 | 200255 | 200806 | + |
| MMSF00249 | *Labrenzia aggregata* | *cobP* | positive | Contig_15_47.504 | 93618 | 94169 | - |
| MMSF00132 | *Labrenzia aggregata* | *cobQ* | positive | Contig_26_56.6932 | 206715 | 208205 | + |
| MMSF00249 | *Labrenzia aggregata* | *cobQ* | positive | Contig_15_47.504 | 86219 | 87709 | - |
| MMSF00132 | *Labrenzia aggregata* | *cobU* | positive | Contig_24_56.1446 | 78969 | 79994 | + |
| MMSF00249 | *Labrenzia aggregata* | *cobU* | positive | Contig_13_45.8339 | 40345 | 41370 | + |
| MMSF00132 | *Labrenzia aggregata* | *cobV* | positive | Contig_24_56.1446 | 77990 | 78862 | - |
| MMSF00249 | *Labrenzia aggregata* | *cobV* | positive | Contig_13_45.8339 | 39366 | 40238 | - |
| MMSF00132 | *Labrenzia aggregata* | *cbiD* | positive | Contig_6_57.2063 | 553941 | 555035 | + |
| MMSF00249 | *Labrenzia aggregata* | *cbiD* | positive | Contig_17_46.7174 | 820470 | 821564 | + |
| MMSF00964 | *Marinobacter salsuginis* | *cbiO* | positive | Contig_36_38.8121 | 420124 | 420720 | + |

**Table S4:** Summary of vitamin B_12_ biosynthesis pathway genes. A “+” indicates the presence of the gene in the respective isolate, whereas a “-“ represents the absence of that gene. Genes in red were not found in any isolate.

| **Isolate** | **Genus Level Classification** | **CobP as indicator** | **Gene Count** | **CobA** | **CobB** | **CobC** | **CobD** | **CobE** | **CobF** | **CobG** |
| --- | --- | --- | --- | --- | --- | --- | --- | --- | --- | --- |
| **MMSF01163** | *Alteromonas* | - | 0 | - | - | - | - | - | - | - |
| **MMSF00257** | *Alteromonas* | - | 0 | - | - | - | - | - | - | - |
| **MMSF00958** | *Alteromonas* | - | 0 | - | - | - | - | - | - | - |
| **MMSF00404** | *Alteromonas* | - | 0 | - | - | - | - | - | - | - |
| **MMSF00132** | *Labrenzia* | + | 18 | **+** | **+** | **+** | **+ x2** | - | - | - |
| **MMSF00249** | *Labrenzia* | + | 18 | **+** | **+** | **+** | **+ x2** | - | - | - |
| **MMSF00964** | *Marinobacter* | - | 2 | - | - | - | - | - | - | - |
| **MMSF01190** | *Marinobacter* | - | 1 | - | - | - | - | - | - | - |
| **MMSF00068** | *Micrococcus* | - | 0 | - | - | - | - | - | - | - |
| **MMSF00107** | *Micrococcus* | - | 0 | - | - | - | - | - | - | - |
| **MMSF00910** | *Winogradskyella* | - | 0 | - | - | - | - | - | - | - |
| **MMSF00046** | *Winogradskyella* | - | 0 | - | - | - | - | - | - | - |

| **Isolate** | **Genus Level Classification** | **CobH** | **CobI** | **CobJ** | **CobK** | **CobL** | **CobM** | **CobN** | **CobO** | **CobP** | **CobQ** |
| --- | --- | --- | --- | --- | --- | --- | --- | --- | --- | --- | --- |
| **MMSF01163** | *Alteromonas* | - | - | - | - | - | - | - | - | - | - |
| **MMSF00257** | *Alteromonas* | - | - | - | - | - | - | - | - | - | - |
| **MMSF00958** | *Alteromonas* | - | - | - | - | - | - | - | - | - | - |
| **MMSF00404** | *Alteromonas* | - | - | - | - | - | - | - | - | - | - |
| **MMSF00132** | *Labrenzia* | **+** | **+** | - | **+** | **+** | **+** | **+** | **+** | **+** | **+** |
| **MMSF00249** | *Labrenzia* | **+** | **+** | - | **+** | **+** | **+** | **+** | **+** | **+** | **+** |
| **MMSF00964** | *Marinobacter* | - | - | - | - | - | - | - | **+** | - | - |
| **MMSF01190** | *Marinobacter* | - | - | - | - | - | - | - | **+** | - | - |
| **MMSF00068** | *Micrococcus* | - | - | - | - | - | - | - | - | - | - |
| **MMSF00107** | *Micrococcus* | - | - | - | - | - | - | - | - | - | - |
| **MMSF00910** | *Winogradskyella* | - | - | - | - | - | - | - | - | - | - |
| **MMSF00046** | *Winogradskyella* | - | - | - | - | - | - | - | - | - | - |

| **Isolate** | **Genus Level Classification** | **CobP** | **CobQ** | **CobR** | **CobS** | **CobT** | **CobU** | **CobV** | **CobW** | **CobX** | **CobY** | **CobZ** | **Cbi** |
| --- | --- | --- | --- | --- | --- | --- | --- | --- | --- | --- | --- | --- | --- |
| **MMSF01163** | *Alteromonas* | - | - | - | - | - | - | - | - | - | - | - | - |
| **MMSF00257** | *Alteromonas* | - | - | - | - | - | - | - | - | - | - | - | - |
| **MMSF00958** | *Alteromonas* | - | - | - | - | - | - | - | - | - | - | - | - |
| **MMSF00404** | *Alteromonas* | - | - | - | - | - | - | - | - | - | - | - | - |
| **MMSF00132** | *Labrenzia* | **+** | **+** | - | - | - | **+** | **+** | - | - | - | - | **cbiD** |
| **MMSF00249** | *Labrenzia* | **+** | **+** | - | - | - | **+** | **+** | - | - | - | - | **cbiD** |
| **MMSF00964** | *Marinobacter* | - | - | - | - | - | - | - | - | - | - | - | **cbiO** |
| **MMSF01190** | *Marinobacter* | - | - | - | - | - | - | - | - | - | - | - | - |
| **MMSF00068** | *Micrococcus* | - | - | - | - | - | - | - | - | - | - | - | - |
| **MMSF00107** | *Micrococcus* | - | - | - | - | - | - | - | - | - | - | - | - |
| **MMSF00910** | *Winogradskyella* | - | - | - | - | - | - | - | - | - | - | - | - |
| **MMSF00046** | *Winogradskyella* | - | - | - | - | - | - | - | - | - | - | - | - |

**Table S5:** Composition of R2A broth adjusted to suit marine bacteria. Final pH = 7.2 +/- 0.2 at 26 °C. R2A broth was made by suspending 43.12 g of combined reagents in 1 L of MilliQ water, dissolving the medium completely, and sterilization by autoclaving at 121˚C for 15 min.

| **Component** | **grams L^–1^** | **Supplier** |
| --- | --- | --- |
| Casein acid hydrolysate | 0.500 | Cat#C0501, Sigma-Aldrich |
| Yeast extract | 0.500 | Cat#LP0021, Oxoid |
| Proteose peptone | 0.500 | Cat#211684, ThermoFisher |
| Dextrose | 0.500 | Cat#G360, PhytoTech Laboratories |
| Starch, soluble | 0.500 | Cat#AJA526, Univar |
| Dipotassium phosphate | 0.300 | Cat#P3786, Sigma-Aldrich |
| Magnesium sulfate | 0.024 | Cat#M2643, Sigma-Aldrich |
| Sodium pyruvate | 0.300 | Cat#P2256, Sigma-Aldrich |
| Red Sea Salt™ | 40.00 | Cat#R11065, Red Sea |

**Table S6:** Single-copy housekeeping genes extracted from the RAST annotations

| **Gene** | **Function** | **NCBI** |
| --- | --- | --- |
| 16S | SSU rRNA ## 16S rRNA, small subunit ribosomal RNA | 16S ribosomal RNA |
| 23S | LSU rRNA ## 23S rRNA, large subunit ribosomal RNA | 23S ribosomal RNA |
| fusA | Translation elongation factor G | elongation factor G |
| gyrB | DNA gyrase subunit B (EC 5.99.1.3) | DNA gyrase subunit B |
| gyrA | DNA gyrase subunit A (EC 5.99.1.3) | DNA gyrase subunit A |
| pyrG | CTP synthase (EC 6.3.4.2) | CTP synthetase |
| lepA | Translation elongation factor LepA | GTP-binding protein LepA |
| recA | RecA protein | recA protein |
| recG | ATP-dependent DNA helicase RecG (EC 3.6.4.12) | ATP-dependent DNA helicase RecG |
| rpoB | DNA-directed RNA polymerase beta subunit (EC 2.7.7.6) | DNA-directed RNA polymerase subunit beta |
| rpoD | RNA polymerase sigma factor RpoD | RNA polymerase sigma factor RpoD |
| atpD | ATP synthase beta chain (EC 3.6.3.14) | F0F1 ATP synthase subunit beta |
| ppK | Polyphosphate kinase (EC 2.7.4.1) | polyphosphate kinase |
| polA | DNA polymerase I (EC 2.7.7.7) | DNA polymerase I |
| pheSa | Phenylalanyl-tRNA synthetase alpha chain (EC 6.1.1.20) | phenylalanyl-tRNA synthetase subunit alpha |
| pheSb | Phenylalanyl-tRNA synthetase beta chain (EC 6.1.1.20) | phenylalanyl-tRNA synthetase subunit beta |
| ileS | Isoleucyl-tRNA synthetase (EC 6.1.1.5) | isoleucyl-tRNA synthetase |
| leuS | Leucyl-tRNA synthetase (EC 6.1.1.4) | leucyl-tRNA synthetase |
| aspS | Aspartyl-tRNA synthetase (EC 6.1.1.12) | aspartyl-tRNA synthetase |
| alaS | Alanyl-tRNA synthetase (EC 6.1.1.7) | alanyl-tRNA synthetase |
| argS | Arginyl-tRNA synthetase (EC 6.1.1.19) | arginyl-tRNA synthetase |
| hisS | Histidyl-tRNA synthetase (EC 6.1.1.21) | histidyl-tRNA synthetase |
| valS | Valyl-tRNA synthetase (EC 6.1.1.9) | valyl-tRNA synthetase |
| srp1 | SSU ribosomal protein S1p | 30S ribosomal protein S1 |
| srp2 | SSU ribosomal protein S2p (SAe) | 30S ribosomal protein S2 |
| srp3 | SSU ribosomal protein S3p (S3e) | 30S ribosomal protein S3 |
| srp4 | SSU ribosomal protein S4p (S9e) | 30S ribosomal protein S4 |
| srp5 | SSU ribosomal protein S5p (S2e) | 30S ribosomal protein S5 |
| srp6 | SSU ribosomal protein S6p | 30S ribosomal protein S6 |
| srp7 | SSU ribosomal protein S7p (S5e) | 30S ribosomal protein S7 |
| srp8 | SSU ribosomal protein S8p (S15Ae) | 30S ribosomal protein S8 |
| srp9 | SSU ribosomal protein S9p (S16e) | 30S ribosomal protein S9 |
| srp10 | SSU ribosomal protein S10p (S20e) | 30S ribosomal protein S10 |
| srp11 | SSU ribosomal protein S11p (S14e) | 30S ribosomal protein S11 |
| srp12 | SSU ribosomal protein S12p (S23e) | 30S ribosomal protein S12 |
| srp13 | SSU ribosomal protein S13p (S18e) | 30S ribosomal protein S13 |
| srp14 | SSU ribosomal protein S14p (S29e) @ SSU ribosomal protein S14p (S29e), zinc-independent | 30S ribosomal protein S14 |
| srp15 | SSU ribosomal protein S15p (S13e) | 30S ribosomal protein S15 |
| srp16 | SSU ribosomal protein S16p | 30S ribosomal protein S16 |
| srp17 | SSU ribosomal protein S17p (S11e) | 30S ribosomal protein S17 |
| srp18 | SSU ribosomal protein S18p @ SSU ribosomal protein S18p, zinc-independent | 30S ribosomal protein S18 |
| srp19 | SSU ribosomal protein S19p (S15e) | 30S ribosomal protein S19 |
| srp20 | SSU ribosomal protein S20p | 30S ribosomal protein S20 |
| srp21 | SSU ribosomal protein S21p | 30S ribosomal protein S21 |
| lrp1 | LSU ribosomal protein L1p (L10Ae) | 50S ribosomal protein L1 |
| lrp2 | LSU ribosomal protein L2p (L8e) | 50S ribosomal protein L2 |
| lrp3 | LSU ribosomal protein L3p (L3e) | 50S ribosomal protein L3 |
| lrp4 | LSU ribosomal protein L4p (L1e) | 50S ribosomal protein L4 |
| lrp5 | LSU ribosomal protein L5p (L11e) | 50S ribosomal protein L5 |
| lrp6 | LSU ribosomal protein L6p (L9e) | 50S ribosomal protein L6 |
| lrp7 | LSU ribosomal protein L7/L12 (P1/P2) | 50S ribosomal protein L7 |
| lrp8 | LSU ribosomal protein L8p | 50S ribosomal protein L8 |
| lrp9 | LSU ribosomal protein L9p | 50S ribosomal protein L9 |
| lrp10 | LSU ribosomal protein L10p (P0) | 50S ribosomal protein L10 |
| lrp11 | LSU ribosomal protein L11p (L12e) | 50S ribosomal protein L11 |
| lrp12 | LSU ribosomal protein L12p | 50S ribosomal protein L12 |
| lrp13 | LSU ribosomal protein L13p (L13Ae) | 50S ribosomal protein L13 |
| lrp14 | LSU ribosomal protein L14p (L23e) | 50S ribosomal protein L14 |
| lrp15 | LSU ribosomal protein L15p (L27Ae) | 50S ribosomal protein L15 |
| lrp16 | LSU ribosomal protein L16p (L10e) | 50S ribosomal protein L16 |
| lrp17 | LSU ribosomal protein L17p | 50S ribosomal protein L17 |
| lrp18 | LSU ribosomal protein L18p (L5e) | 50S ribosomal protein L18 |
| lrp19 | LSU ribosomal protein L19p | 50S ribosomal protein L19 |
| lrp20 | LSU ribosomal protein L20p | 50S ribosomal protein L20 |
| lrp21 | LSU ribosomal protein L21p | 50S ribosomal protein L21 |
| lrp22 | LSU ribosomal protein L22p (L17e) | 50S ribosomal protein L22 |
| lrp23 | LSU ribosomal protein L23p (L23Ae) | 50S ribosomal protein L23 |
| lrp24 | LSU ribosomal protein L24p (L26e) | 50S ribosomal protein L24 |
| lrp25 | LSU ribosomal protein L25p | 50S ribosomal protein L25 |
| lrp26 | LSU ribosomal protein L26p | 50S ribosomal protein L26 |
| lrp27 | LSU ribosomal protein L27p | 50S ribosomal protein L27 |
| lrp28 | LSU ribosomal protein L28p | 50S ribosomal protein L28 |
| lrp29 | LSU ribosomal protein L29p (L35e) | 50S ribosomal protein L29 |
| lrp30 | LSU ribosomal protein L30p (L7e) | 50S ribosomal protein L30 |
| lrp31 | LSU ribosomal protein L31p @ LSU ribosomal protein L31p, zinc-independent | 50S ribosomal protein L31 |
